# Supplementary material for: Inhibition of lysyl oxidases synergizes with 5-azacytidine to restore erythropoiesis in myelodysplastic and myeloid malignancies
Source: Nat Commun. 2023 Mar 17;14:1497. doi: 10.1038/s41467-023-37175-8 (PMC10023686; doi:10.1038/s41467-023-37175-8)
Supplement: Supplementary file 1 — Supplementary Information [file 41467_2023_37175_MOESM1_ESM.pdf]

Supplementary Table 1. Patient cohort for RT-qPCR analysis

No.	Subtype WHO 2016	IPSS-R for MDS and CPSS for CMML	Cell types analyzed			
P3*	CMML I	Intermediate-1	MNC	CD71+		CD34+
P21*	MDS-RS-MLD	Intermediate		CD71+	MSC	CD34+
P22*	MDS-RS-MLD	Low	MNC	CD71+	MSC	
P26*	MDS with isolated del(5q)	Intermediate-1	MNC	CD71+	MSC	
P32	PMF	*NA*			MSC	
P33	PMF	*NA*	MNC	CD71+	MSC	CD34+
P34	CMML II	Intermediate-1	MNC	CD71+	MSC	
P35	MDS-EB I	Intermediate		CD71+	MSC	CD34+
P36	MDS-MLD	Very low	MNC	CD71+	MSC	
P37	MPN, unclassifiable	*NA*	MNC		MSC	CD34+
P38	tCMML 0	Intermediate-1	MNC	CD71+	MSC	CD34+
P39	CMML I	Intermediate-2	MNC	CD71+	MSC	CD34+
P40	CMML I	Intermediate-1	MNC	CD71+	MSC	
P41	MDS-EB II	High	MNC	CD71+	MSC	CD34+
P42	CMML I	Intermediate-2	MNC		MSC	CD34+
P43	MDS-MLD	Very low			MSC	
P44	CMML I	Low	MNC	CD71+	MSC	CD34+
P45	MDS-MLD	Intermediate		CD71+	MSC	CD34+
P46	PMF	*NA*	MNC	CD71+		
P47	Secondary MF after PV	*NA*	MNC	CD71+		CD34+

*Samples of P3, P21, P22 and P26 were obtained from the time points of bone marrow aspiration, which were different from that shown in Supplementary Table 6. Abbreviations: *NA*, not applicable; RT-qPCR, quantitative reverse transcription PCR; WHO, World Health Organization; IPSS-R, revised international prognostic scoring system; CPSS, CMML-specific prognostic scoring; MDS-RS-MLD, MDS with multiple lineage dysplasia and ring sideroblasts; MDS-MLD, MDS with multilineage dysplasia; and MDS-EB, MDS with excess blasts; MF, myelofibrosis; PV, polycythemia vera.

Supplementary Table 2. Healthy cohort for RT-qPCR analysis

No.	Age	Gender	Cell type analyzed
HY8	68	F	MSC
HY9	64	M	MSC
HY10	77	M	MSC
HY11	69	M	MSC
HY12	76	F	MSC
HY13	74	F	MSC
HY14	75	F	MSC
HY15	79	F	MSC
HY16	65	F	MSC
Median age, years	74 (64-79)		

Abbreviations: HY, healthy; RT-qPCR, quantitative reverse transcription PCR; MSC, mesenchymal stem cell.

Supplementary Table 3. Characteristics of patient and healthy cohorts for LOX and LOXL2 concentration and activity determination in BM plasma

	**Patient cohort**	**Healthy cohort**
Number of patient/healthy cohort	94*	15
Male	60	5
Female	34	10
Sample number	109*	15
Median age for samples (years)	73 (43-88)	25 (21-79)
WHO 2016 diagnosis of samples		
MDS	86	
MPN	4	
CMML	7	
AML-MRC	12	
BMF degree of samples		
BMF0	40	
BMF0-1	5	
BMF1	31	
BMF1-2	9	
BMF2	17	
BMF2-3	1	
BMF3	6	

*Eleven patients had two BM plasma samples collected at time-points with different BMF degree. Two patients had three BM plasma samples collected at time-points with different BMF degrees. Abbreviations: LOX/LOXL, lysyl oxidase and lysyl-oxidase-like; BM, bone marrow; WHO, World Health Organization; MDS, MDS, myelodysplastic syndrome; MPN, myeloproliferative neoplasm; CMML, chronic myelomonocytic leukemia; AML-MRC, acute myeloid leukemia with myelodysplasia-related changes; BMF, bone marrow fibrosis.

Supplementary Table 4. Characteristics of patients and healthy donors for CellTiter-Glo cell viability assay

No.	Subtype WHO 2016	IPSS-R for MDS and CPSS for CMML	Cell types analyzed	
P5*	MDS-EB II	Unknown		MSC
P13	MDS-EB II	Very high	CD34+ cells	
P23*	AML-MRC	*NA*	CD34+ cells	MSC
P31	AML-MRC	*NA*	CD34+ cells	MSC
P34*	CMML II	Intermediate 1	CD34+ cells	MSC
P48	CMML I	Low	CD34+ cells	MSC
P49	MDS-MLD	Low	CD34+ cells	MSC
P50	MDS-MLD	Very high	CD34+ cells	
P51	MDS-EB II	High	CD34+ cells	
P52	MDS-EB I	Very high		MSC
P53	MDS-MLD	Intermediate		MSC
HY5	-	-	CD34+ cells	MSC
HY6	-	-	CD34+ cells	MSC
HY7	-	-	CD34+ cells	MSC

*Samples of P5, P23 and P34 were obtained from the time point of bone marrow aspiration which were different from that shown in Supplementary Table 1 (P34) or Supplementary Table 6 (P5, P23). Abbreviations: *NA*, not applicable; WHO, World Health Organization; IPSS-R, revised international prognostic scoring system; MDS, myelodysplastic syndrome; CMML, chronic myelomonocytic leukemia; CPSS, CMML-specific prognostic scoring; AML-MRC, acute myeloid leukemia with myelodysplasia-related changes; MDS-MLD, MDS with multilineage dysplasia; MDS-EB, MDS with excess blasts; MSC, mesenchymal stem cell.

Supplementary Table 5. Patient characteristics for LOX/LOXL activity inhibiting assay in MSCs

No.	Subtype WHO 2016	IPSS-R for MDS and CPSS for CMML
P32	PMF	*NA*
P42	CMML I	Intermediate 2
P54	MDS-EB II	Very high
P55	AML-MRC	*NA*
P56	MDS-MLD	Low

Abbreviations: *NA*, not applicable; LOX/LOXL, lysyl oxidase and lysyl-oxidase-like; MSC, mesenchymal stem cell; WHO, World Health Organization; IPSS-R, revised international prognostic scoring system; MDS, myelodysplastic syndrome; CMML, chronic myelomonocytic leukemia; CPSS, CMML-specific prognostic scoring; PMF, primary myelofibrosis; MDS-EB, MDS with excess blasts; AML-MRC, acute myeloid leukemia with myelodysplasia-related changes; MDS-MLD, MDS with multilineage dysplasia.

Supplementary Table 6. Patient characteristics for MSCs and CD34+ HSPCs for co-culture assays and murine PDX experiments

No.	Subtype WHO 2016*	IPSS-R for MDS and CPSS for CMML*	Karyotype*	Hemoglobin, g/dl*	BMF grade*	Prior Therapy†	Experiments
P1	MDS-MLD	Intermediate-1	46,XY[20]	9.9	3	No specific therapy	*In vitro*
P2	CMML II	Intermediate-2	46,XY.ish del(4)(q24q24)(*TET2*-)[10]/46,XY[10]	10.7	1	Hydroxyurea	*In vitro*
P3	CMML I	Intermediate-1	46,XX[20]	8.7	0	No specific therapy	*In vitro* and PDX
P4	MDS-RS-SLD	Low	46,XY[20]	7.8	1-2	No specific therapy	*In vitro*
P5	AML-MRC	*NA*	46,XY,+1,der(1;16)(q10;p10)[18]/46,XY[2]	8.3	Unknown	No specific therapy	*In vitro*
P6	MDS-EB II	High	46,XY,del(3)(q13q25)[20]	6.8	2	4 cycles of 5-AZA	*In vitro*
P7	AML-MRC	*NA*	46,XX[21]	9.1	0	1 cycle of 5-AZA	*In vitro*
P8	MDS-RS-MLD	Low	46,XX[20]	8.2	2	Hydroxyurea + EPO	*In vitro*
P9	CMML II	High	44,XX,der(3)del(3p)del(3q),del(5q),-6,der(12)t(12;17),-der(17),+min[20]	8.9	0	Hydroxyurea	*In vitro*
P10, time1	MDS-EB I	High	46,XY,t(1;3)(p36;q21)[17]/47,XY,t(1;3)(p36;q21),+13[2]/46,XY[3]	8.2	0	No specific therapy	*In vitro*
P10, time2	MDS-EB I	High	46,XY,t(1;3)(p36;q21)[19]/46,XY[1]	8.1	0	13 cycles of 5-AZA + Venetoclax	*In vitro*
P11	CMML II	High	46,XY,r(6)(p21q26)[20]	9.7	1	EPO	*In vitro* and PDX
P12	MDS-MLD	Intermediate	46,XY,t(3;5)(q25;q35)	8.7	1	No specific therapy	*In vitro*
P13	MDS-EB II	Very high	46,XY,+1,del(5)(q13q34),dic(16;17)(q11;p11)[6]/46,XY,+1,del(5)(q13q34),dic(16;17)(q11;p11),der(18)t(3;18)(q21;q22)[10]/46,XY,+1,del(5)(q13q34),der(6)t(6;11)(q24;q13),dic(16;17)(q11;p11)[3]/46,XY[1]	10	0	1 cycle of 5-AZA + Venetoclax	*In vitro*
P14	MDS-MLD	Very low	46,XY[20]	13.9	2	No specific therapy	*In vitro*

P15	PMF/MPN-U	*NA*	46,XX[20]	14.1	0	Hydroxyurea; Phlebotomies	*In vitro*
P16	MDS-EB II	Very high	47,XY+8[4]/46,XY[21]	7.2	0	No specific therapy	*In vitro*
P17	MDS, NOS	Intermediate	46,XY[20]	8.6	1	Iron chelation	*In vitro*
P18	CMML I	Unknown	Unknown	8.6	1-2	No specific therapy	*In vitro* and PDX
P19	CMML I	Intermediate-1	46,XY[25]	7.6	Unknown	No specific therapy	*In vitro*
P20	MDS-MLD	Intermediate-2	46,XX,der(2)(X;2)(p21;q37),der(10)t(X;10)(p11;p14),del(20)(q11q13)[11]/46,XX,der(2)(X;2)(p21;q37),dup(8)(q21q23)[5]/46,XX[4]	10.5	1	Unknown	*In vitro*
P21	MDS-RS-MLD	High	46,XY,del(5)(q14),der(11)t(11;16)(q22;q12),der(16)t(11;16)(q23;p13)t(5;16)(q14;q12)[16]/46,XY[4]	7.8	0	Hydroxyurea	*In vitro*
P22	MDS-RS-MLD	Low	46,XX[20]	9.5	2	Asunercept; EPO	*In vitro*
P23, time1	MDS-MLD	Low	46,XX[20]	7.9	0	EPO	*In vitro*
P23, time2	AML-MRC	*NA*	47,XX,+6[16]/46,XX[9]	10.2	1	EPO, 1 cycle of 5-AZA	*In vitro*
P24	MDS-RS-MLD	Very low	46,XY[20]	7.9	1	EPO, G-CSF	*In vitro*
P25	MDS-EB II	High	46,XX[25]	12.0	3	No specific therapy	*In vitro*
P26	MDS with isolated del(5q)	Intermediate-1	45,XY,dic(7;22)(q11;p11)[2]/46,XY[18]	11.7	1-2	Deferoxamine; Lenalidomide; Dexamethasone	*In vitro*
P27	MDS/MPN-U	Low	46,XY,i(17)(q10)[21]	8.6	0	Hydroxyurea	*In vitro*
P28	CMML I	Intermediate-1	46,XY[20]	8.3	1	Dexamethasone	*In vitro*
P29	CMML I	Intermediate-2	47,XY,+21[2]/46,XY[19]	8.8	0	No specific therapy	*In vitro*
P30	MDS-EB II	Very high	44,XY,der5,t(5;12)(p14q11),-7[7]/ 46,XY[14]	7.6	1	No specific therapy	*In vitro* and PDX
P31	AML-MRC	*NA*	46,XY[18]	8.7	0	No specific therapy	*In vitro*

P32	PMF	*NA*	46,XX[12]	8.2	3	Hydroxyurea	PDX
P33	PMF	*NA*	45,X,-Y[20]	8.4	1	No specific therapy	PDX
HY1	Healthy	*NA*	*NA*	14.7	*NA*	*NA*	*In vitro*
HY2	Healthy	*NA*	*NA*	14.2	*NA*	*NA*	*In vitro*
HY3	Healthy	*NA*	*NA*	14.0	*NA*	*NA*	*In vitro*
HY4	Healthy	*NA*	*NA*	13.5	*NA*	*NA*	*In vitro*
HY5	Healthy	*NA*	*NA*	14.8	*NA*	*NA*	*In vitro*
HY6	Healthy	*NA*	*NA*	14.2	*NA*	*NA*	*In vitro*
HY7	Healthy	*NA*	*NA*	15.0	*NA*	*NA*	*In vitro*

*Clinical information applies for the date of sample collection.

†Therapy information before the date of sample collection.

Abbreviations: *NA*, not applicable; M, male; F, female; WHO, World Health Organization; IPSS-R, revised international prognostic scoring system; CPSS, CMML-specific prognostic scoring; MDS-MLD, MDS with multilineage dysplasia; MDS-RS-SLD, MDS with single lineage dysplasia and ring sideroblasts; MDS-EB, MDS with excess blasts; MDS-RS-MLD, MDS with multiple lineage dysplasia and ring sideroblasts; PMF/MPN-U, PMF/MPN-unclassifiable; MDS NOS, MDS unclassified; MDS/MPN-U, MDS/MPN-unclassifiable; and G-CSF, granulocyte-colony stimulating factor; EPO, erythropoietin.

Supplementary Table 7. The list of ECM proteins affected by PXS-5505 treatment in P3

**Peak Name**	**Group**	**Mean, untreated**	**Mean, PXS-5505**	**Delta (mean PXS-5505 - mean untreated)**	**Fold Change (mean PXS-5505 / mean untreated)**
sp|P51884|LUM_HUMAN	Lumican OS=Homo sapiens OX=9606 GN=LUM PE=1 SV=2	5,569,790	2,736,431	-2,833,360	0.49
sp|Q14767|LTBP2_HUMAN	Latent-transforming growth factor beta-binding protein 2 OS=Homo sapiens OX=9606 GN=LTBP2 PE=1 SV=3	1,643,178	2,202,696	559,518	1.34
sp|P12107|COBA1_HUMAN	Collagen alpha-1(XI) chain OS=Homo sapiens OX=9606 GN=COL11A1 PE=1 SV=4	5,052,762	3,148,913	-1,903,849	0.62
sp|Q15582|BGH3_HUMAN	Transforming growth factor-beta-induced protein ig-h3 OS=Homo sapiens OX=9606 GN=TGFBI PE=1 SV=1	15,149,777	16,629,255	1,479,478	1.10
sp|P09486|SPRC_HUMAN	SPARC OS=Homo sapiens OX=9606 GN=SPARC PE=1 SV=1	3,411,238	2,222,788	-1,188,450	0.65
sp|P08123|CO1A2_HUMAN	Collagen alpha-2(I) chain OS=Homo sapiens OX=9606 GN=COL1A2 PE=1 SV=7	114,906,681	178,815,068	63,908,387	1.56

Unit: Mass spectrometry signal intensity counts.
The data were normalized to the total mass spectrometry signal.

Supplementary Table 8. The list of ECM proteins affected by PXS-5505 treatment in P10

**Peak Name**	**Group**	**Mean, untreated**	**Mean, PXS-5505**	**Delta (mean PXS-5505 - mean untreated)**	**Fold Change (mean PXS-5505 / mean untreated)**
sp|Q6UVK1|CSPG4_HUMAN	Chondroitin sulfate proteoglycan 4 OS=Homo sapiens OX=9606 GN=CSPG4 PE=1 SV=2	651,029	844,335	193,307	1.30
sp|P07585|PGS2_HUMAN	Decorin OS=Homo sapiens OX=9606 GN=DCN PE=1 SV=1	356,457	52,447	-304,010	0.15
sp|P10915|HPLN1_HUMAN	Hyaluronan and proteoglycan link protein 1 OS=Homo sapiens OX=9606 GN=HAPLN1 PE=2 SV=2	376,311	555,073	178,762	1.48
sp|P21810|PGS1_HUMAN	Biglycan OS=Homo sapiens OX=9606 GN=BGN PE=1 SV=2	1,326,147	335,732	-990,415	0.25
sp|P35052|GPC1_HUMAN	Glypican-1 OS=Homo sapiens OX=9606 GN=GPC1 PE=1 SV=2	244,683	311,415	66,733	1.27
sp|P09486|SPRC_HUMAN	SPARC OS=Homo sapiens OX=9606 GN=SPARC PE=1 SV=1	168,476	56,868	-111,608	0.34

Unit: Mass spectrometry signal intensity counts.
The data were normalized to the total mass spectrometry signal.

Supplementary Table 9. The list of ECM proteins affected by PXS-5505 treatment in P11

Peak Name	Group	Mean, untreated	Mean, PXS-5505	Delta (mean PXS-5505 - mean untreated)	Fold Change (mean PXS-5505 / mean untreated)
sp|P35052|GPC1_HUMAN	Glypican-1 OS=Homo sapiens OX=9606 GN=GPC1 PE=1 SV=2	1,569,760	981,283	-588,478	0.63
sp|P09486|SPRC_HUMAN	SPARC OS=Homo sapiens OX=9606 GN=SPARC PE=1 SV=1	9,308,637	7,256,552	-2,052,085	0.78
sp|P12107|COBA1_HUMAN	Collagen alpha-1(XI) chain OS=Homo sapiens OX=9606 GN=COL11A1 PE=1 SV=4	26,207,968	10,816,428	-15,391,540	0.41
sp|P07996|TSP1_HUMAN	Thrombospondin-1 OS=Homo sapiens OX=9606 GN=THBS1 PE=1 SV=2	146,263,393	126,666,749	-19,596,644	0.87
sp|P05997|CO5A2_HUMAN	Collagen alpha-2(V) chain OS=Homo sapiens OX=9606 GN=COL5A2 PE=1 SV=3	102,456,757	47,572,744	-54,884,013	0.46
sp|P08572|CO4A2_HUMAN	Collagen alpha-2(IV) chain OS=Homo sapiens OX=9606 GN=COL4A2 PE=1 SV=4	5,737,476	4,166,338	-1,571,138	0.73
sp|P35442|TSP2_HUMAN	Thrombospondin-2 OS=Homo sapiens OX=9606 GN=THBS2 PE=1 SV=2	5,177,688	4,480,451	-697,237	0.87
sp|Q6UVK1|CSPG4_HUMAN	Chondroitin sulfate proteoglycan 4 OS=Homo sapiens OX=9606 GN=CSPG4 PE=1 SV=2	5,109,622	3,865,456	-1,244,166	0.76
sp|Q99715|COCA1_HUMAN	Collagen alpha-1(XII) chain OS=Homo sapiens OX=9606 GN=COL12A1 PE=1 SV=2	39,741,332	27,864,781	-11,876,551	0.70
sp|P08123|CO1A2_HUMAN	Collagen alpha-2(I) chain OS=Homo sapiens OX=9606 GN=COL1A2 PE=1 SV=7	346,665,506	485,421,441	138,755,935	1.40
sp|P20908|CO5A1_HUMAN	Collagen alpha-1(V) chain OS=Homo sapiens OX=9606 GN=COL5A1 PE=1 SV=3	34,829,057	16,185,038	-18,644,018	0.46

sp|P51884|LUM_HUMAN	Lumican OS=Homo sapiens OX=9606 GN=LUM PE=1 SV=2	8,622,715	4,043,685	-4,579,030	0.47
sp|Q07092|COGA1_HUMAN	Collagen alpha-1(XVI) chain OS=Homo sapiens OX=9606 GN=COL16A1 PE=1 SV=2	2,420,436	1,721,635	-698,802	0.71
sp|Q15582|BGH3_HUMAN	Transforming growth factor-beta-induced protein ig-h3 OS=Homo sapiens OX=9606 GN=TGFBI PE=1 SV=1	37,651,070	46,895,807	9,244,737	1.25
sp|Q96CG8|CTHR1_HUMAN	Collagen triple helix repeat-containing protein 1 OS=Homo sapiens OX=9606 GN=CTHRC1 PE=1 SV=1	1,731,179	2,112,431	381,252	1.22
sp|P07585|PGS2_HUMAN	Decorin OS=Homo sapiens OX=9606 GN=DCN PE=1 SV=1	12,602,545	11,226,031	-1,376,514	0.89

Unit: Mass spectrometry signal intensity counts.
The data were normalized to the total mass spectrometry signal.

Supplementary Figure 1

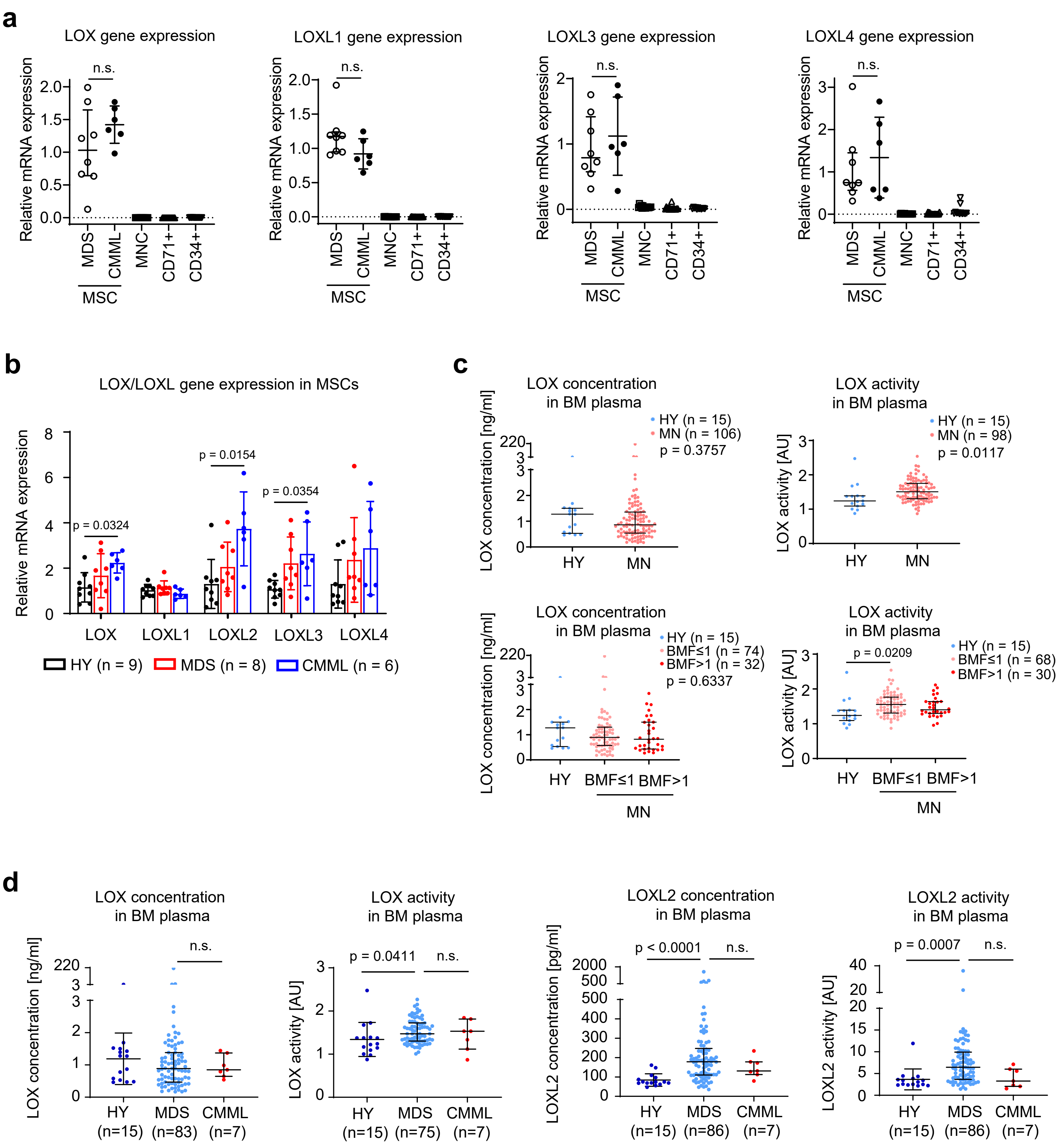

Supplementary Figure 1*. LOX/LOXL* expression and activity in MN.
a, Comparison of *LOX/LOXL* gene expression in different BM fractions of MN patients. *LOX*, *LOXL1* and *LOXL3-4* gene expression in MSCs (n=14), mononuclear cells (MNCs, n=15), CD71+ (n=16) and CD34+ cells (n=12) of n=20 MN patients was assessed using quantitative reverse transcription-PCR (RT-qPCR). The data are median±IQR. Statistical significance was analyzed using two-sided Mann-Whitney U test.
b, *LOX/LOXL* mRNA expression in MSCs is shown separately for HY (n=9), MDS (n=8) and CMML (n=6). The data are median±IQR.
c, The association of LOX enzyme concentration and activity in MN with BMF grade information. LOX concentration and activity were measured in BM plasma samples of HY control and MN patients. The background level of LOX activity (signal-to-noise) was controlled by BAPN treatment. The data for median±IQR are indicated. Statistical significance was analyzed using two-sided Mann-Whitney U-test (top) or Kruskal-Wallis test with Dunn's multiple comparisons (bottom).
d, LOX and LOXL2 activity is shown separately for HY, MDS and CMML patients. The data are median±IQR. For **b** and **d**, statistical significance was analyzed using Kruskal-Wallis test with Dunn's multiple comparisons. For all statistical analyses, p values < 0.05 indicated statistical significance. Source data are provided as a Source Data file.
Abbreviation: n.s., not statistically significant.

Supplementary Figure 2

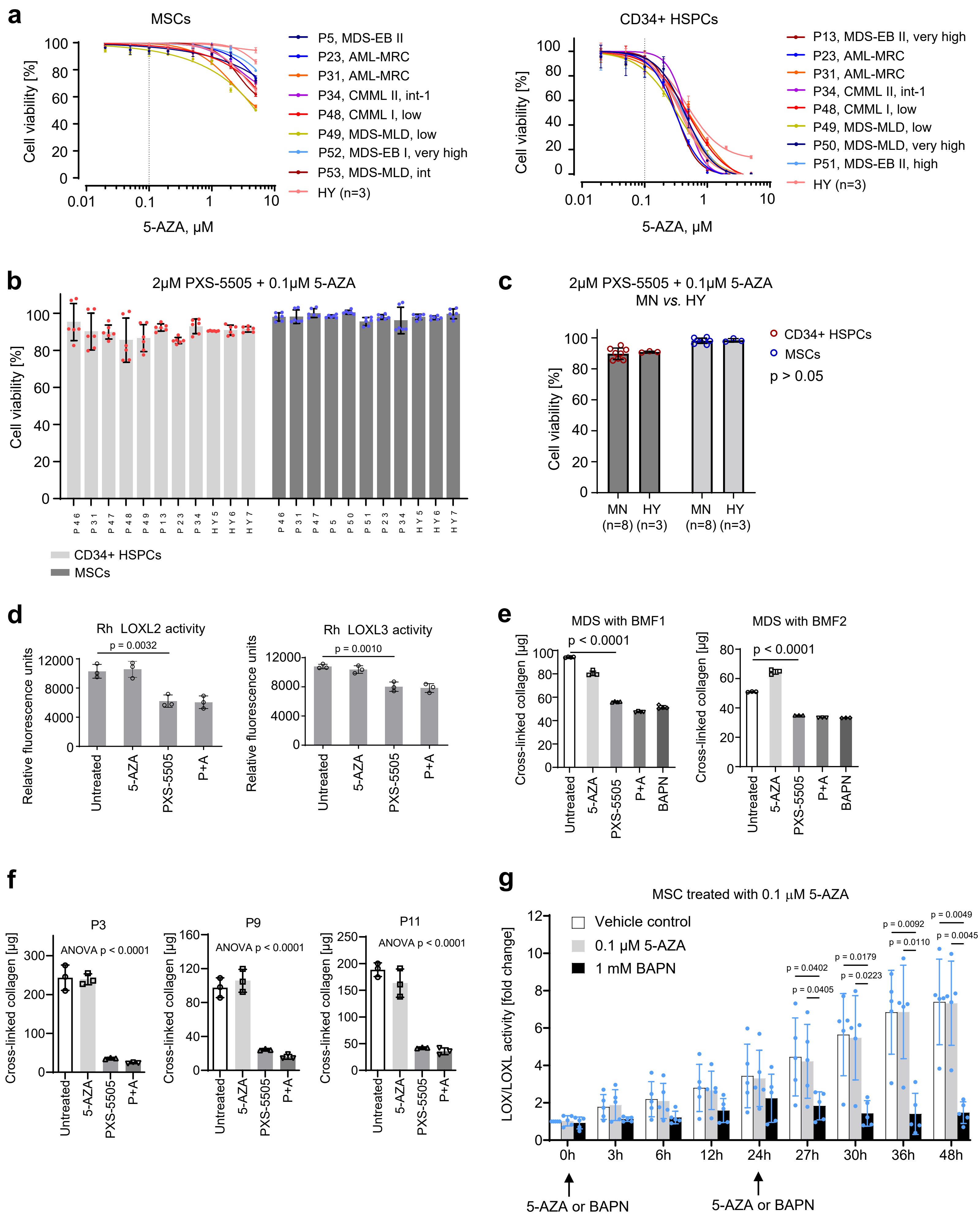

Supplementary Figure 2. The effects of PXS-5505 on cell viability, LOX/LOXL activity and collagen production.

a, The viability of BM MSCs and CD34+ HSPCs of n=11 MN patients (n=8 for MSCs and n=8 for HSPCs; see Supplementary Table 4) and n=3 aged-matched HY exposed to the daily treatment with increasing concentrations of 5-AZA was determined using CellTiter-Glo assay after 4 days of incubation. The data are mean±SD of 5 or 6 cell culture replicates.

b, The viability of MSCs and CD34+ HSPCs in the presence of 2µM PXS-5505 + 0.1µM 5-AZA (P+A) was assessed for patients analyzed in (**a**) and **Fig. 1h, i** using CellTiter-Glo assay. MSCs were pretreated with PXS-5505 daily for 7 days, followed by daily treatment of P+A for 4 days. CD34+ HSPCs were treated with P+A daily for 4 days. The data are mean±SD of 6 cell culture replicates.

c, Summarized data from (**b**) are shown as mean±SD. Statistical significance between HY and MN was tested using two-sided unpaired Student's t test.

d, Recombinant human (rh) LOXL2 or LOXL3 enzymes (R&D Systems, LOXL2: 2639-AO-010, LOXL3: 6069-AO-010) at the concentration of 5µg/ml were incubated with 0.1µM 5-AZA, 2µM PXS-5505 or P+A for 40 minutes, 37ºC and enzymatic activity was measured using Lysyl Oxidase Activity Assay Kit (Fluorometric) (Abcam, ab112139). The data are mean±SD of triplicates.

e, MSC-derived fibroblasts of n=2 MDS patients with BMF were treated daily for 7 days with 0.1µM 5-AZA, 2µM PXS-5505, P+A or 1mM β-aminopropionitrile (BAPN). Cross-linked collagen production by fibroblasts was determined using Sircol Soluble Collagen Assay kit (Biocolor, S1000). The data are mean±SD of fibroblast culture quadruplicates (left) and triplicates (right).

f, Autologous MSC/HSPC co-culture was performed as shown in Fig. 2a for n=3 cases (P3, P9 and P11 shown in **Supplementary Table 6**). After washing out HSPCs from MSCs, cross-linked collagen production by MSCs was determined using Sircol Soluble Collagen Assay kit. The data are mean±SD of co-culture triplicates.

g, *Ex-vivo* expanded MSCs were treated using 0.1 µM 5-AZA or 1mM BAPN every 24h and pan-LOX/LOXL activity in supernatants of MSC culture was measured using the Lysyl Oxidase Activity Assay Kit. Combined data for n=5 MN patients are presented as mean of fold changes±SD. Statistical significance for each time-point was tested using RM one-way ANOVA with Tukey's multiple comparisons.

Statistical significance in **d-f** was determined using ordinary one-way ANOVA with Tukey's multiple comparisons. For all analyses, p values < 0.05 indicated statistical significance. Source data are provided as a Source Data file.

Supplementary Figure 3

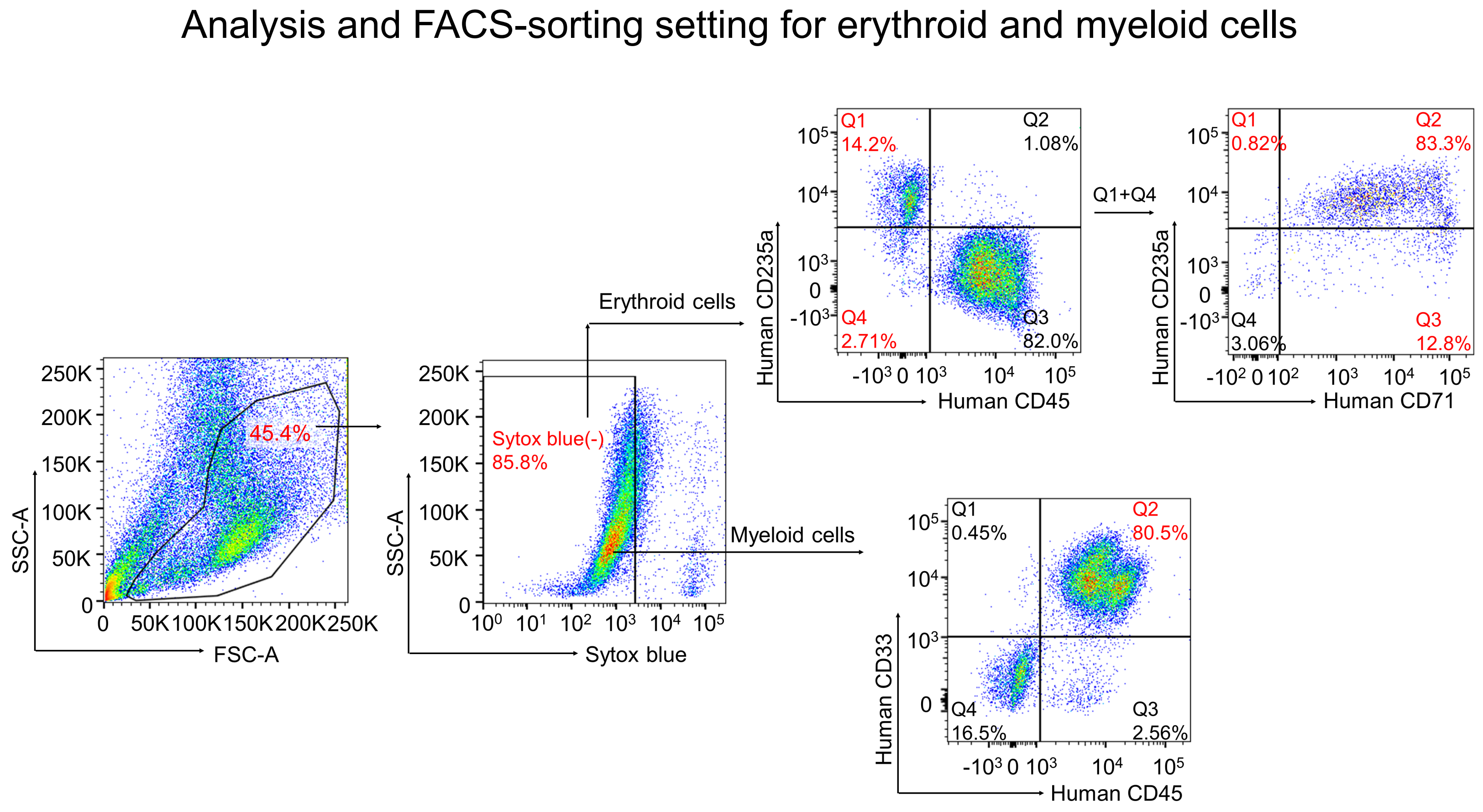

Supplementary Figure 3. Gating strategy for the detection of erythroid (CD235a+CD45-) and myeloid (CD45+CD33+) cells in bulk cellular outputs of CFU assays. FACS, Fluorescence-activated Cell Sorting.

Supplementary Figure 4

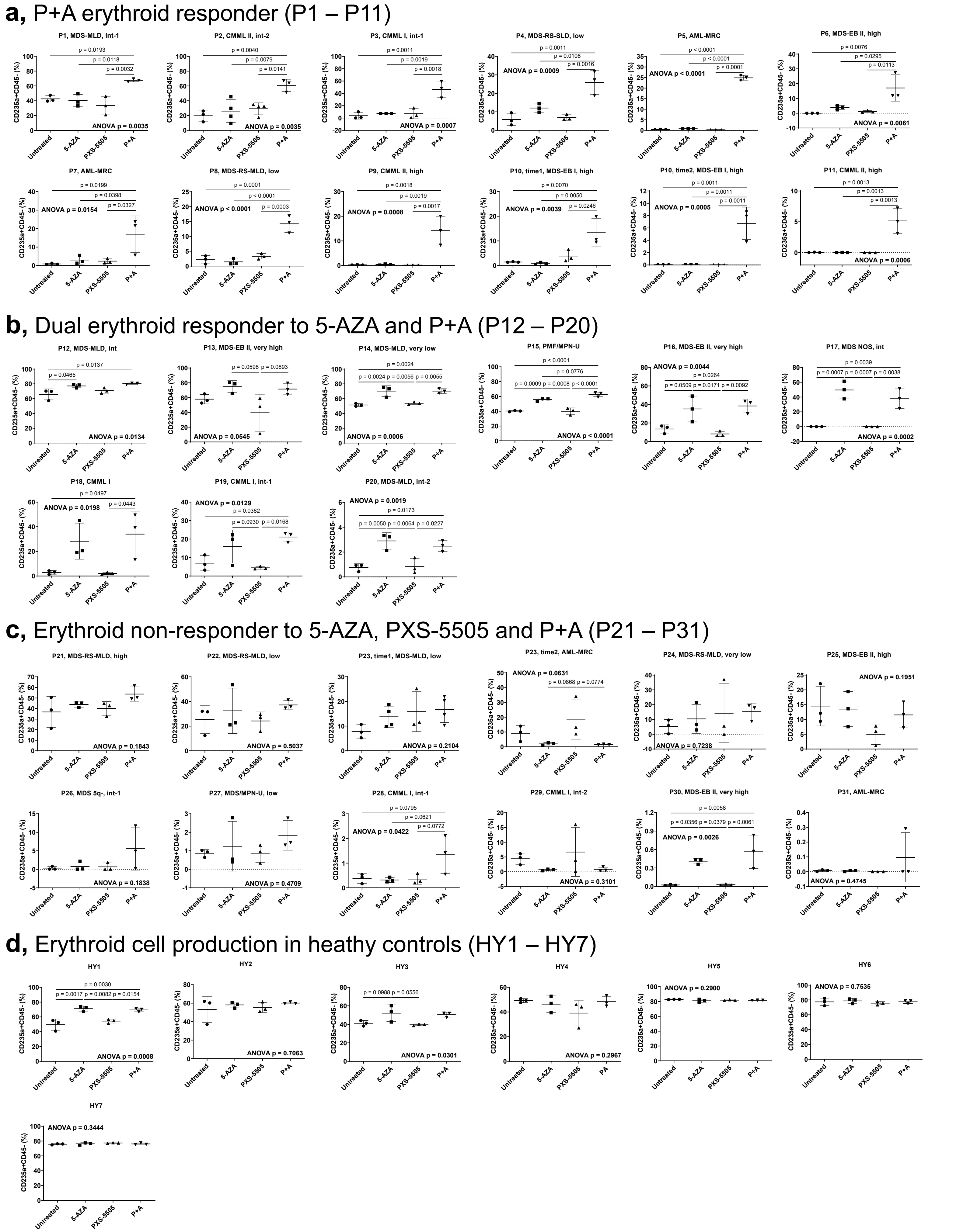

Supplementary Figure 4. Treatment-associated changes in erythroid differentiation of BM CD34+ HSPCs co-cultured with autologous MSCs.

Autologous BM MSC/CD34+ HSPC co-cultures were treated with 5-AZA, PXS-5505 or P+A followed by the assessment of CFU bulk cells for the presence of CD235a+CD45- erythroid progenitors using flow cytometry. Individual data for n=31 MN patients and n=7 HY controls are shown. P10 and P23 had samples from two different time points of bone marrow aspiration.

a, P1-P11 were P+A erythroid responders, identified as a subgroup with improved erythroid production in P+A but not in any of the single substances ($p<0.05$); n=12 biological samples from n=11 MN patients.

b, P12-P20 were dual erythroid responders to 5-AZA and P+A, showing similarly increased percentage of erythroid progenitors in 5-AZA and P+A arms (5-AZA *versus* P+A, $p>0.05$). P13, P16, P18 and P19 were regarded as dual erythroid responders due to a substantial improvement of erythroid production after 5-AZA or P+A treatment, although statistical significance was not reached due to the data heterogeneity; n=9 biological samples from n=9 MN patients.

c, The remaining patients (P21-P31) were regarded as erythroid non-responders; n=12 biological samples from n=11 MN patients.

d, Percentages of CD235a+CD45- erythroid cells are shown for n=7 HY controls.

The data are mean±SD of independent MSC/HSPC co-culture triplicates or quadruplicates per treatment arm. Statistical significance was assessed using ordinary one-way ANOVA with Tukey's multiple comparisons. P values < 0.05 indicated statistical significance.

Abbreviations: MDS-EB, MDS with excess blasts; MDS-RS-SLD, MDS with single lineage dysplasia and ring sideroblasts; AML-MRC, acute myeloid leukemia with myelodysplasia-related changes; MDS-RS-MLD, MDS with multiple lineage dysplasia and ring sideroblasts; MDS-MLD, MDS with multilineage dysplasia; PMF/MPN-U, PMF/MPN-unclassifiable; MDS NOS, MDS unclassified; MDS/MPN-U, MDS/MPN unclassified; int, intermediate.

Source data are provided as a Source Data file.

Supplementary Figure 5

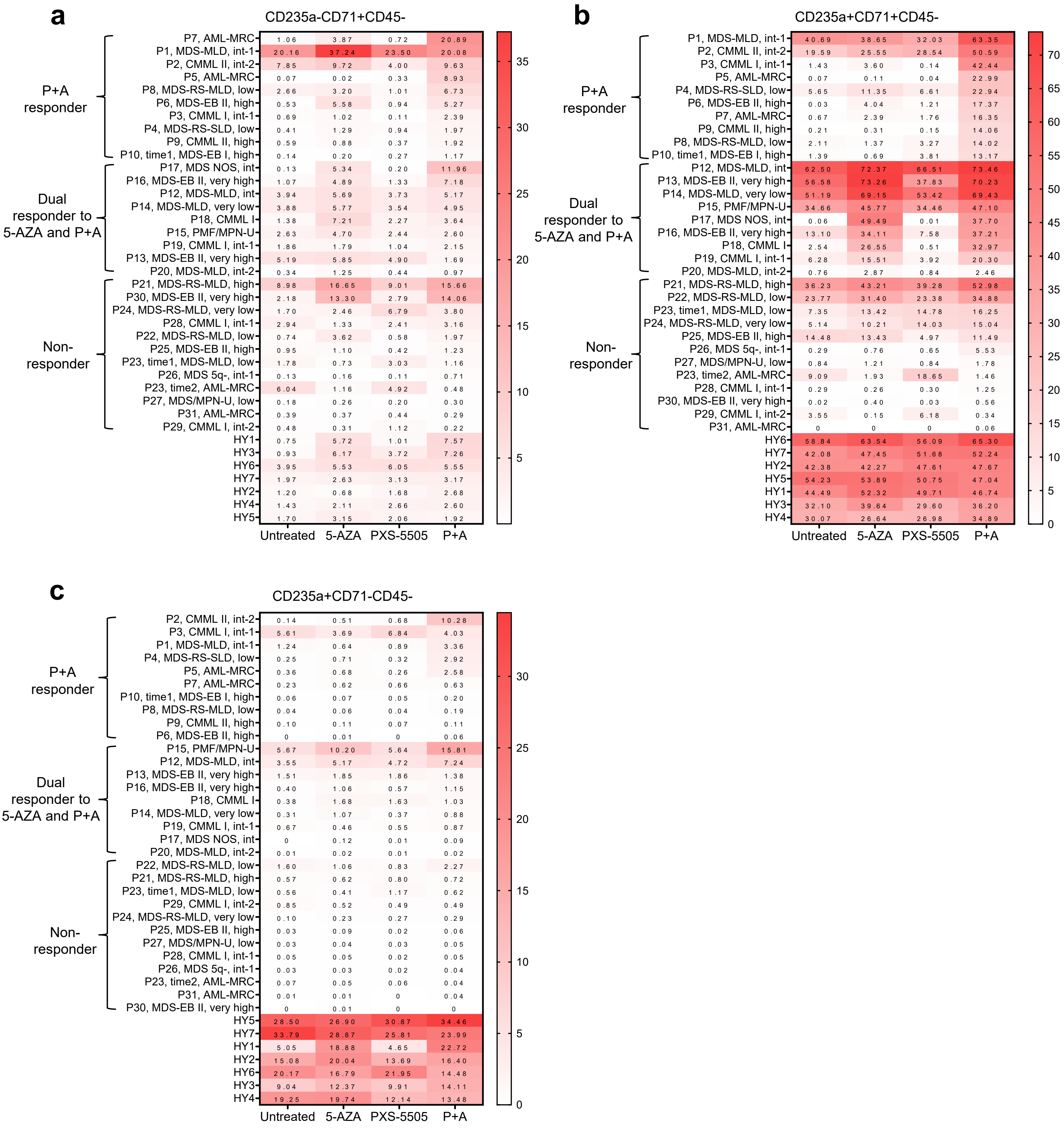

Supplementary Figure 5. Treatment-associated changes in different stages of erythroid differentiation of BM CD34+ HSPCs co-cultured with autologous MSCs.
BM MSC/CD34+ HSPC co-cultures were treated with 5-AZA, PXS-5505 or P+A followed by the assessment of CFU bulk cellular outputs for CD235a, CD71 and CD45 markers by flow cytometry. Heatmaps show percentages of early stage CD235a-CD71+CD45- erythroid progenitors (**a**), intermediate stage CD235a+CD71+CD45- erythroid progenitors (**b**) and late stage CD235a+CD71-CD45- erythroid progenitors (**c**) for n=30 MN patients and n=7 HY controls. Source data are provided as a Source Data file.

Supplementary Figure 6

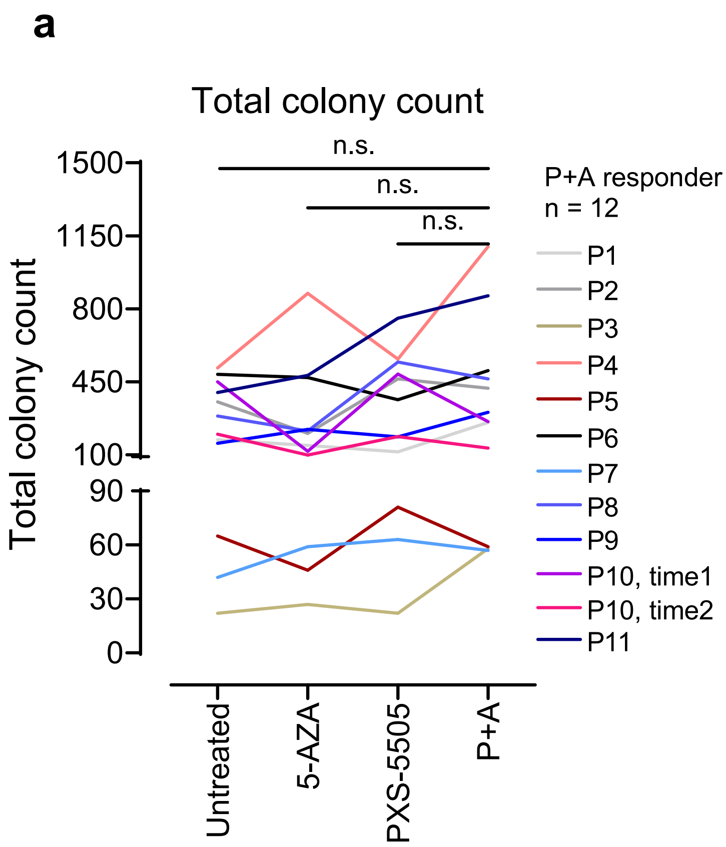

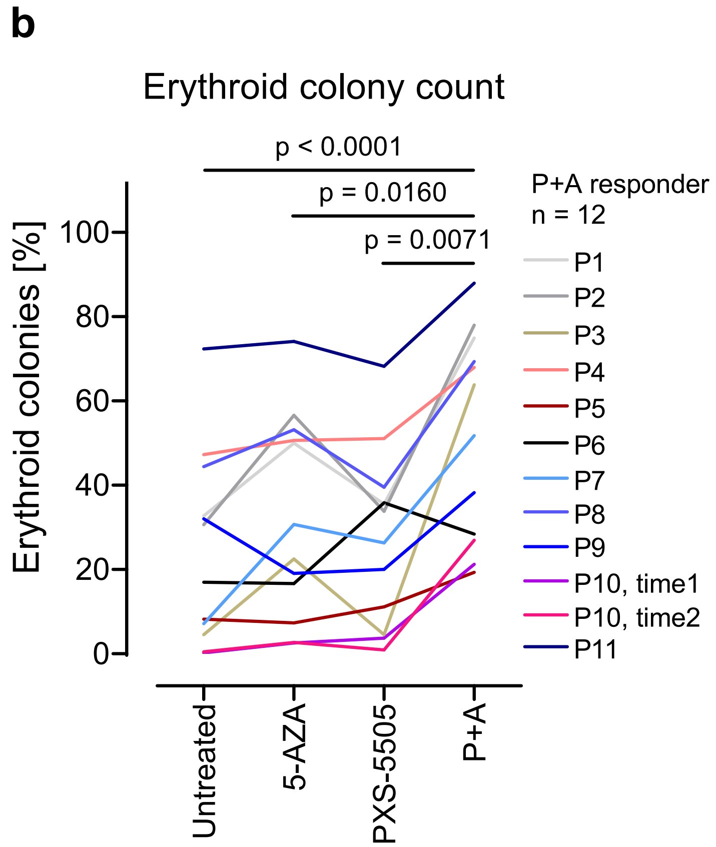

Supplementary Figure 6. CFU colony count in P+A erythroid responders.
a, Depicts the total colony counts of P+A erythroid responders from **Fig. 2e**. n.s. – not significant.
b, The CFU assay results of P+A erythroid responders are presented as a percentage of erythroid colonies among total colonies.
For **a** and **b**, Friedman test with Dunn's multiple comparisons was used for statistical analysis. P values < 0.05 indicated statistical significance. Source data are provided as a Source Data file.

Supplementary Figure 7

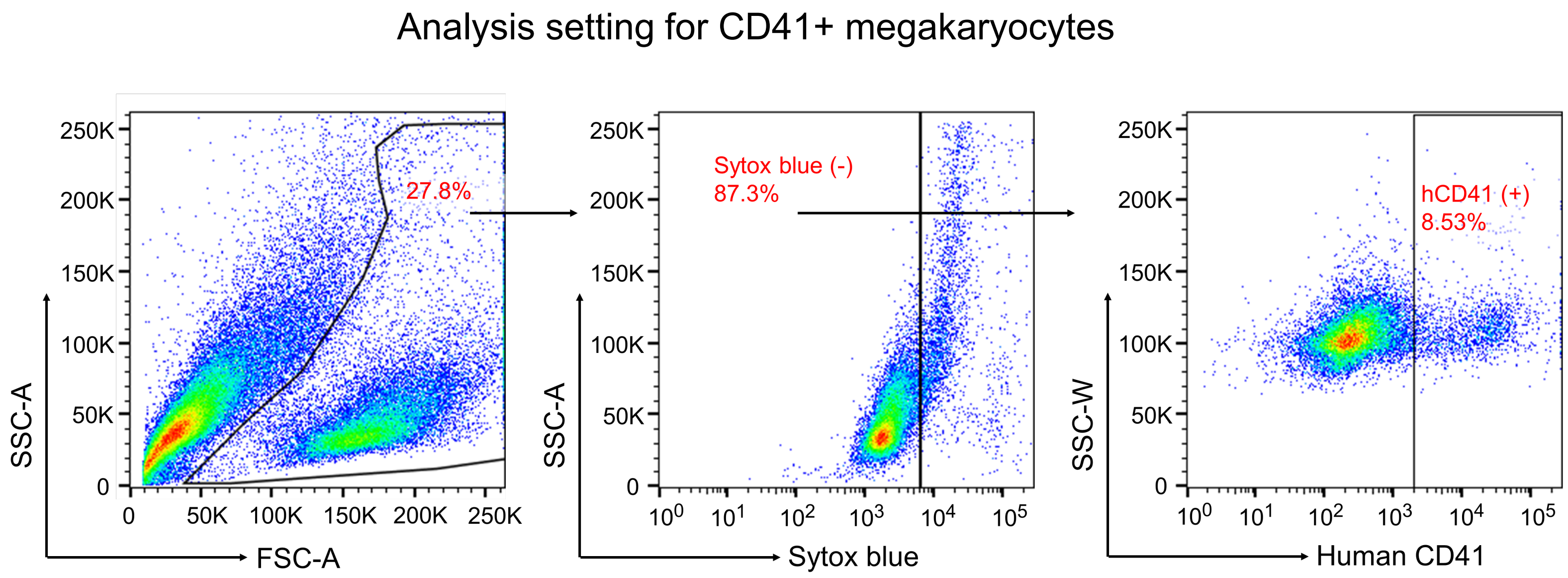

Supplementary Figure 7. Gating strategy for the detection of CD41+ megakaryocytes (MK) in *in-vitro* MK differentiation assay.

Supplementary Figure 8

a, P+A megakaryocytic responder

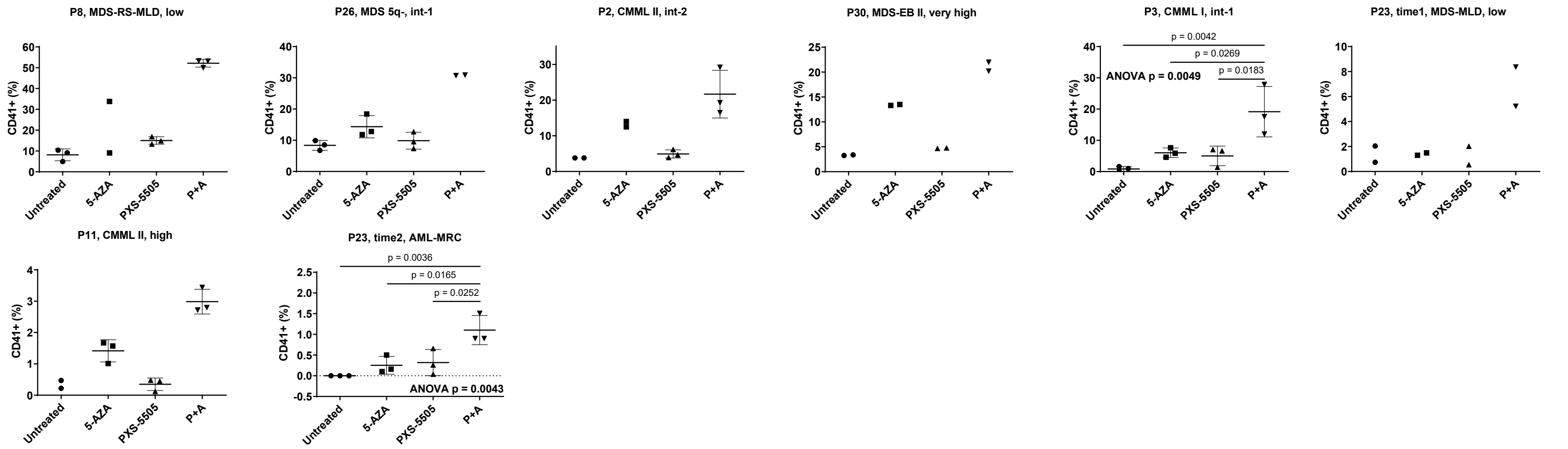

b, Dual megakaryocytic responder to 5-AZA and P+A

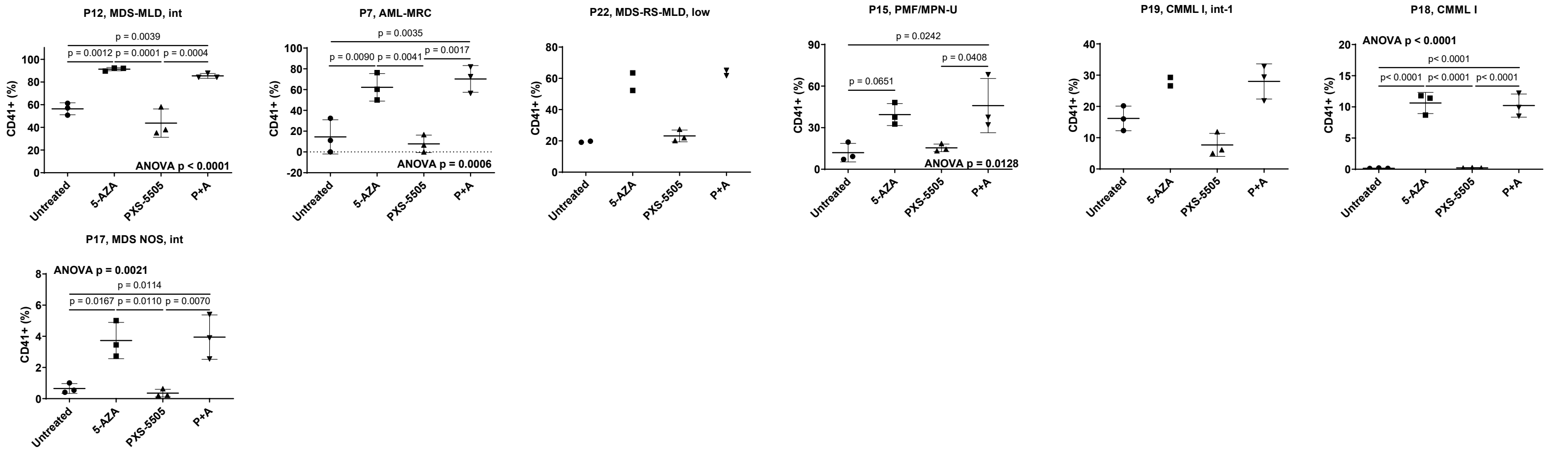

c, Megakaryocytic non-responder to 5-AZA, PXS-5505 and P+A

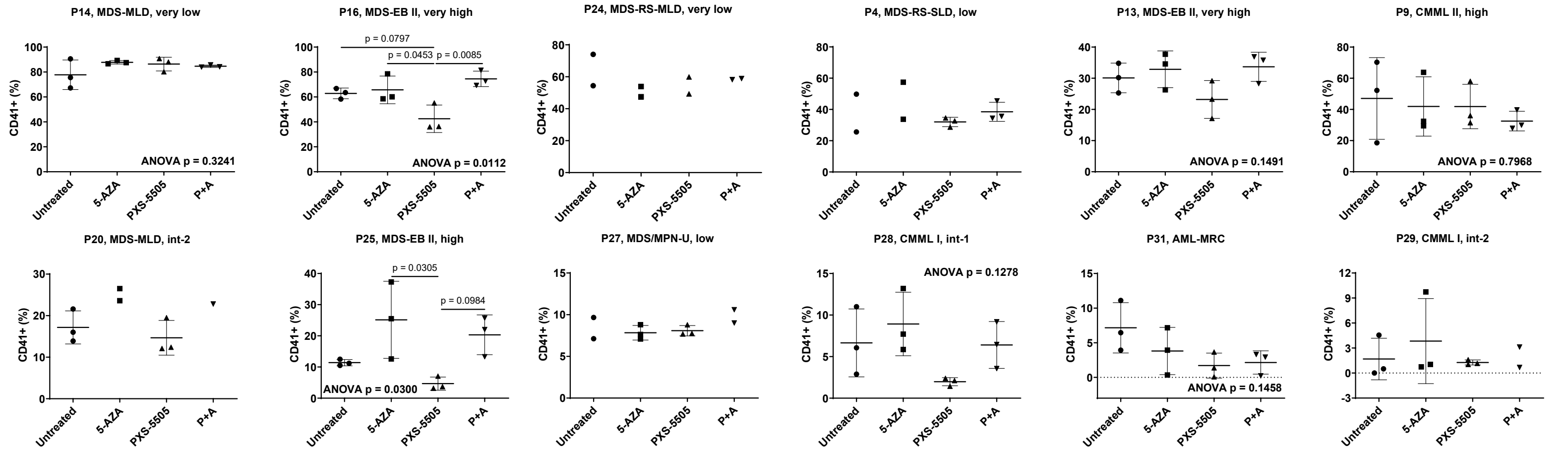

d, Megakaryocyte production in heathy controls

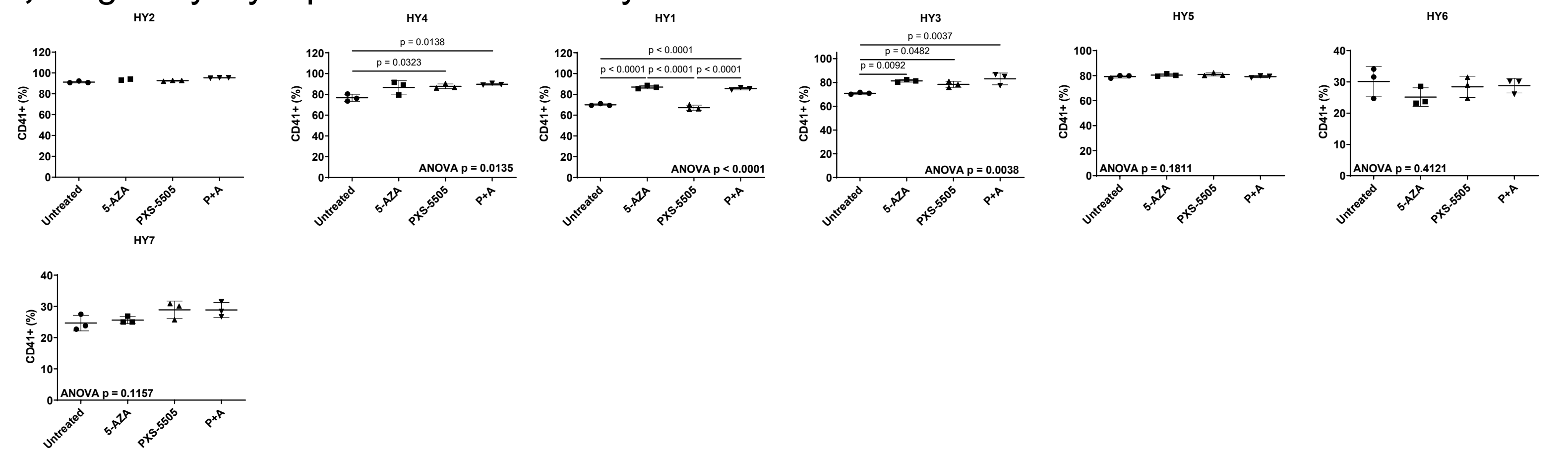

Supplementary Figure 8. Treatment-associated changes in megakaryocyte (MK) differentiation of BM CD34+ HSPCs co-cultured with autologous MSCs.

Autologous BM MSC/CD34+ HSPC co-cultures were treated with 5-AZA, PXS-5505 or P+A followed by the culture of CD34+ HSPCs in the presence of Stemspan Megakaryocyte Expansion Supplement (StemCell Technologies, 02696) for 21 days. The presence of CD41+ MK was analyzed using flow cytometry. Individual data for n=26 MN patients and n=7 HY controls are shown. P23 had samples from two different time points of bone marrow aspiration as explained in Supplementary Figure 4. We defined an *in-vitro* “megakaryocytic response” as a significant increase of percentage of CD41+ MK in 5-AZA, PXS-5505 or P+A arms as compared to vehicle control ($p<0.05$).

a, P+A megakaryocytic responders (n=8 biological samples from n=7 MN patients) were identified as a subgroup with improved MK production in P+A but not in any of the single substances as shown in P3 and P23 (time 2) ($p<0.05$). P8, P26, P2, P30, P23 (time 1) and P11 were regarded as P+A megakaryocytic responders due to substantially increased MK production after P+A treatment as compared to other arms, although only duplicates were done for some treatment arms due to the limited availability of CD34+ HSPCs.

b, Dual megakaryocytic responders to 5-AZA and P+A treatment (n=7 biological samples from n=7 MN patients) showed similarly increased percentage of MK in 5-AZA and P+A arms as compared to untreated arm (5-AZA *versus* P+A, $p>0.05$). P15 and P19 were regarded as dual megakaryocytic responders due to substantially increased MK production after 5-AZA treatment as compared to untreated arm, although statistical significance was not reached due to the data heterogeneity. P22 and P12 were regarded as dual megakaryocytic responders due to substantially increased MK production, although only duplicates were done for some treatment arms due to the limited availability of CD34+ HSPCs.

c, The remaining patients (n=12 biological samples from n=12 MN patients) were regarded as megakaryocytic non-responders after treatment.

d, Percentages of CD41+ MK are shown for n=7 HY controls.

The data are mean±SD of independent MSC/HSPC co-culture duplicates or triplicates per treatment arm. Statistical significance was assessed using ordinary one-way ANOVA with Tukey’s multiple comparisons. P values < 0.05 indicated statistical significance. Abbreviations are explained in **Supplementary Figure 4**. Source data are provided as a Source Data file.

Supplementary Figure 9

Analysis and sorting setting for human CD45+ cells in murine PDX model

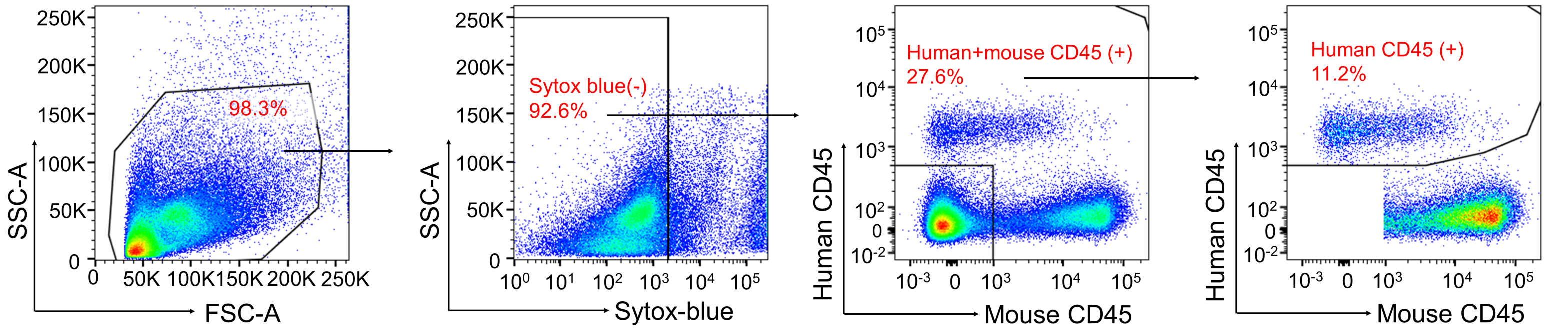

Supplementary Figure 9. Gating strategy for human CD45+ cells from murine PDX model.

Supplementary Figure 10

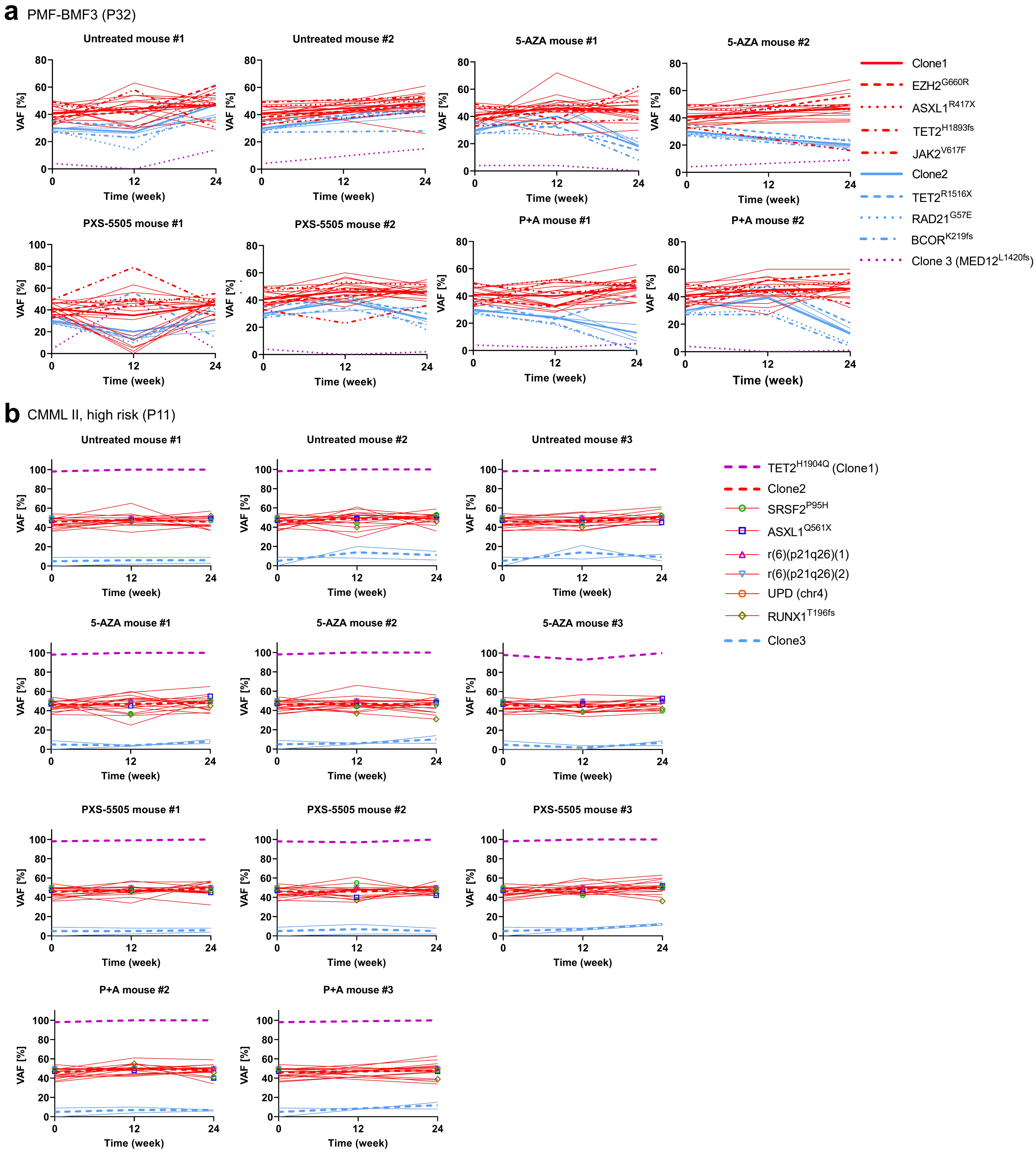

Supplementary Figure 10. Clonal dynamics of patient xenografts (P32 and P11) in the course of *in-vivo* treatment. BM HSPCs + autologous MSCs of P32 (**a**) and P11 (**b**) were transplanted into NSG mice and VAFs (%) of mutations were assessed in initial BM MNC (week 0), xenografts before treatment start (week 12) and at the end of treatment (week 24) using the whole exome sequencing. Clonal composition of xenografted samples was reconstructed using the bioinformatic tool SciClone package (version 1.1). Source data are provided as a Source Data file.

Supplementary Figure 11

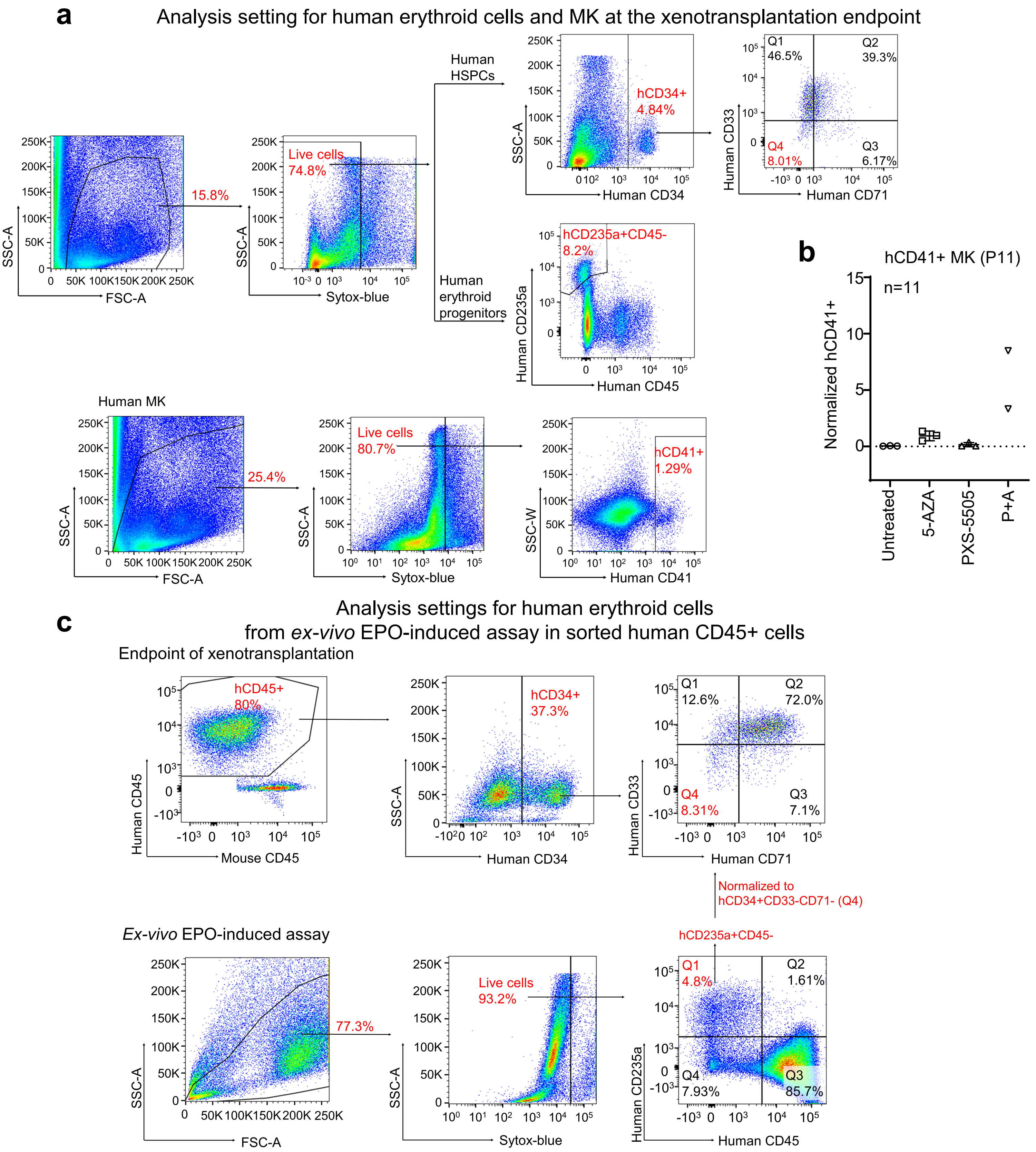

Supplementary Figure 11. Flow cytometry gating strategies and endpoint assessment of MK in PDX.
a, Gating strategy for the identification of human CD34+CD71-CD33- HSPCs, CD235a+CD45- erythroid progenitors and CD41+ MK in the treatment endpoint of murine PDX model.
b, The percentages of human CD41+ MK in n=11 PDX mice were assessed in mouse BM using flow cytometry for P11. The data were normalized to the percentages of human CD34+CD33-CD71- HSPCs in the mouse BM. The data are presented as mean±SD. Source data are provided as a Source Data file.
c, Gating strategy for the identification of patient CD235a+CD45- erythroid cells in *ex-vivo* EPO-induced assay and data normalization.

Supplementary Figure 12

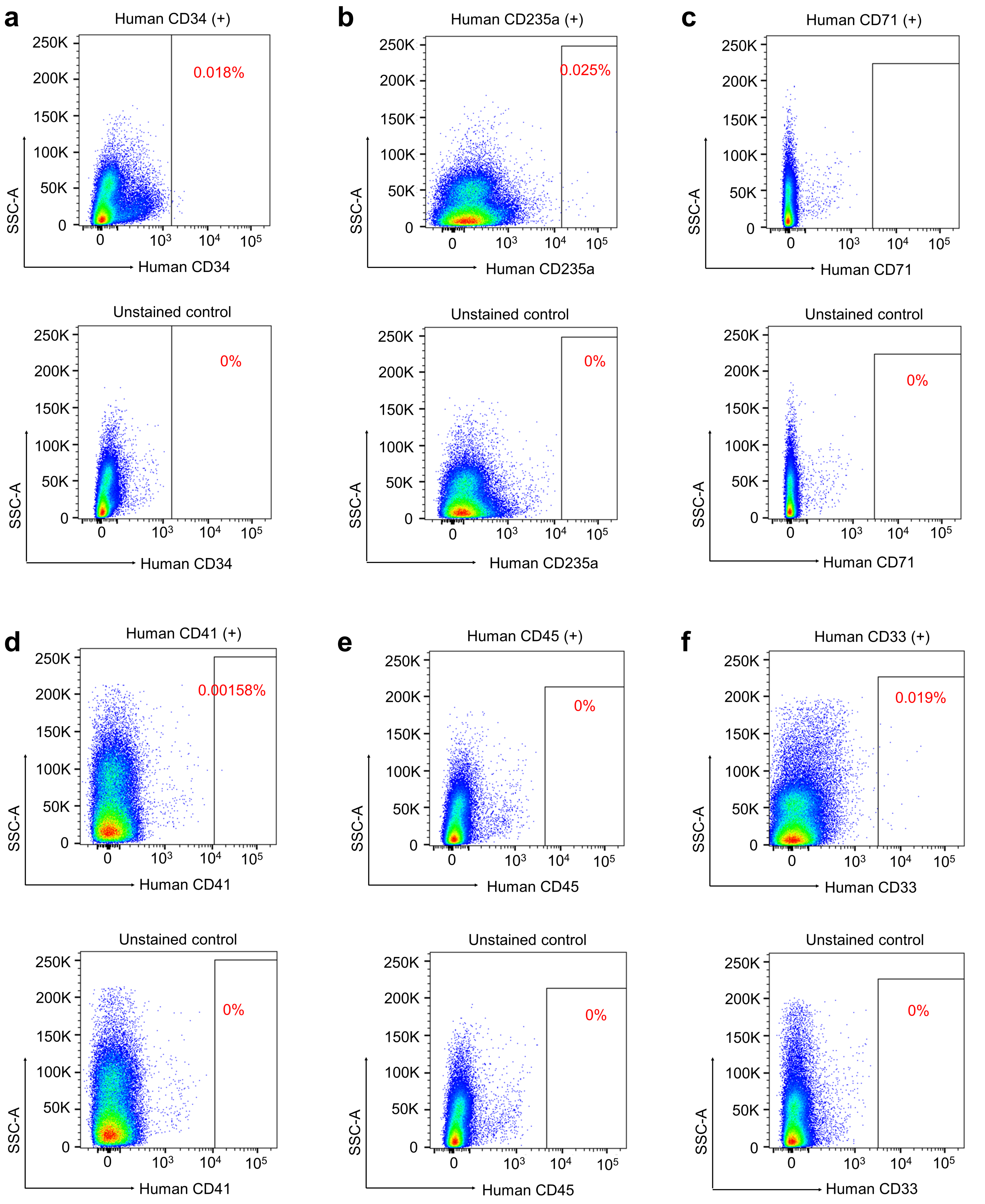

Supplementary Figure 12. Antibody cross-reactivity assessment. Whole BM was isolated from non-transplanted NSG female mouse and labeled using human-specific antibodies to CD34 (**a**), CD235a (**b**), CD71 (**c**), CD41 (**d**), CD45 (**e**) and CD33 (**f**). Percentages of antibody cross-reactivity with mouse cells were defined after gating on unstained controls.

Supplementary Figure 13

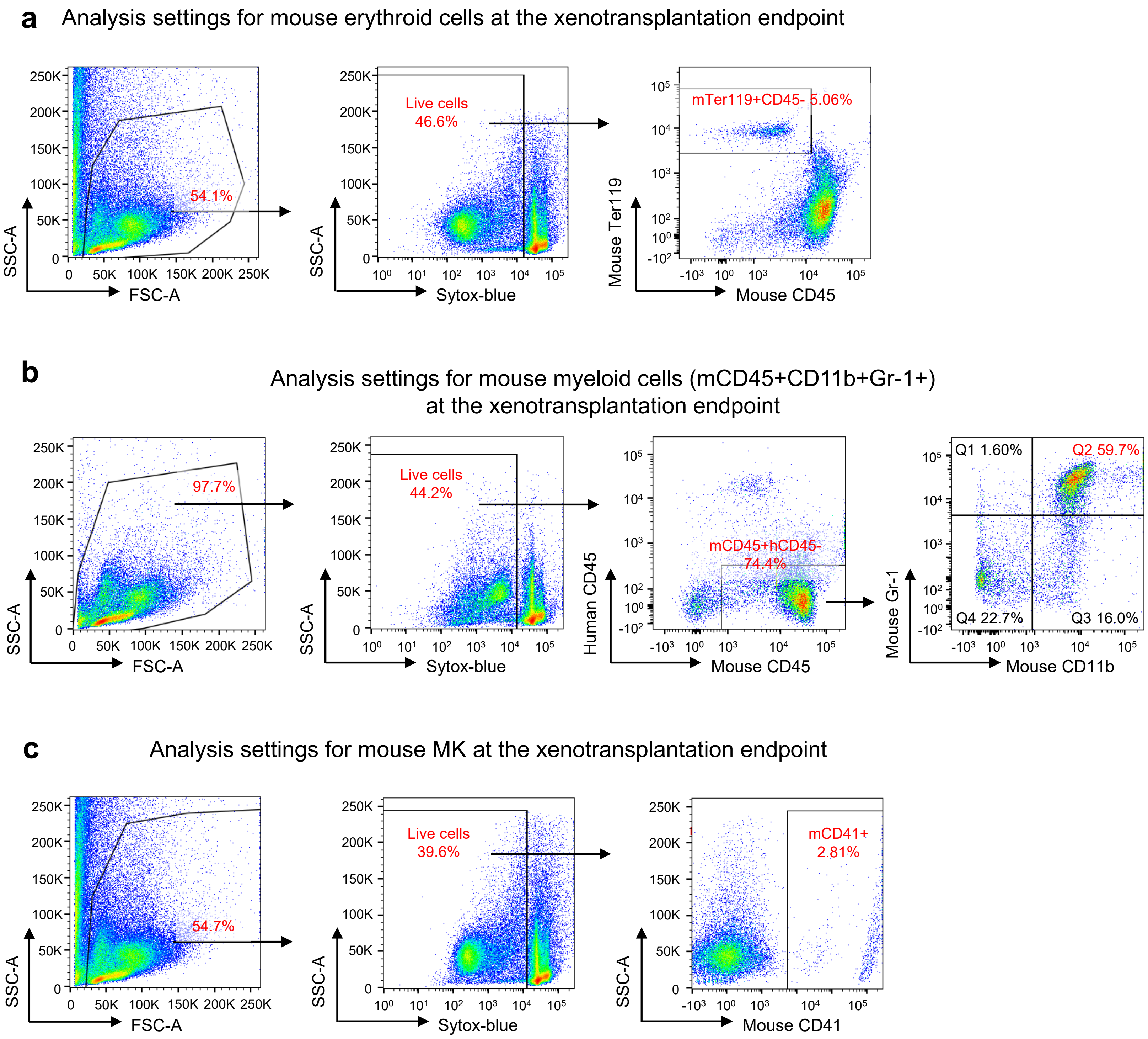

Supplementary Figure 13. Flow cytometry gating strategies for the assessment of mouse hematopoiesis in PDX models.

a, Gating strategy for the identification of mouse Ter119+CD45- erythroid cells at the treatment endpoint of PDX models.

b, Gating strategy for the identification of mouse CD45+CD11b+Gr-1+ myeloid cells at the treatment endpoint of PDX models.

c, Gating strategy for the identification of mouse CD41+ MK at the treatment endpoint of PDX models.

Supplementary Figure 14

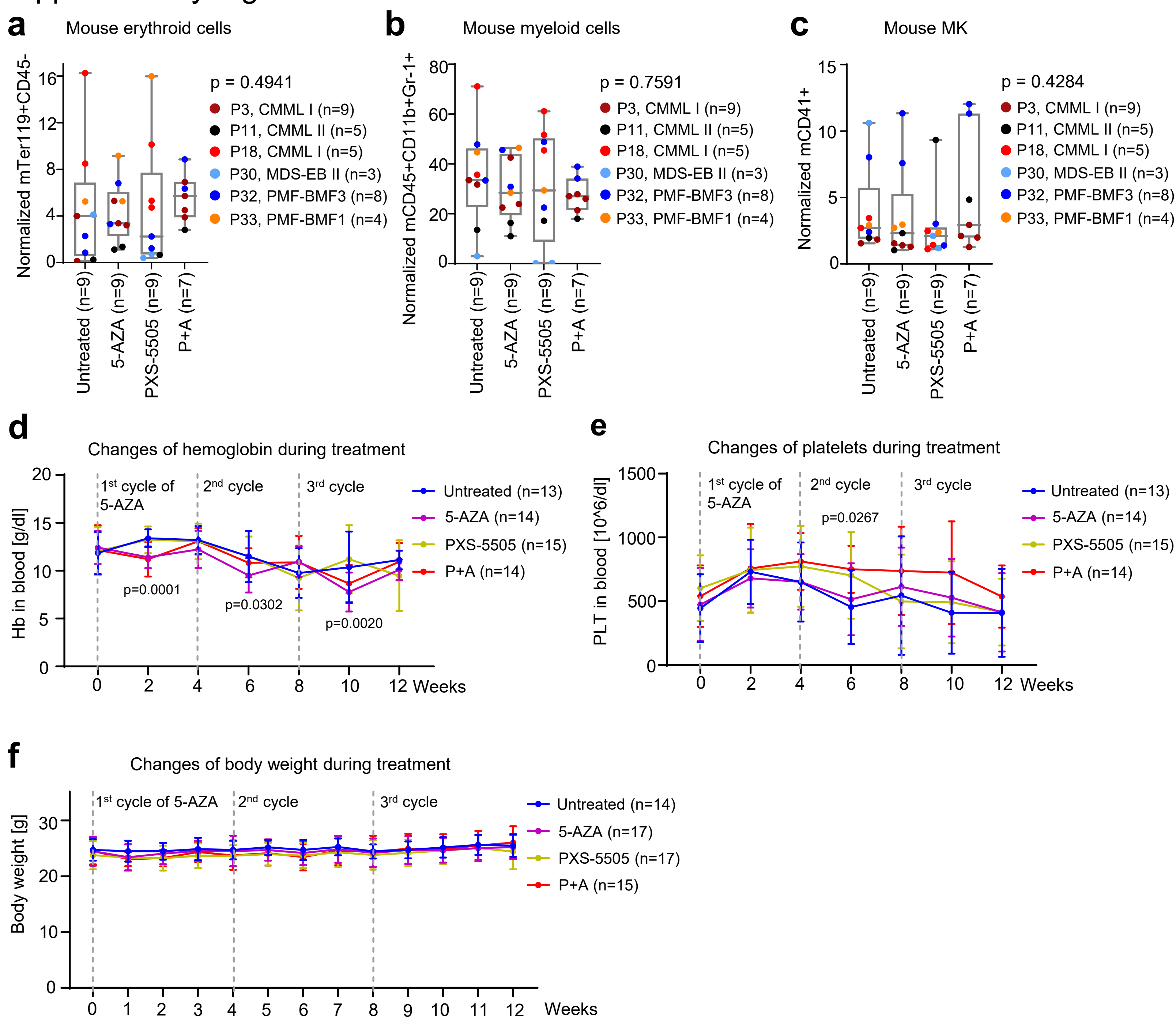

Supplementary Figure 14. PXS-5505 and P+A treatments do not affect mouse hematopoiesis and body weight in PDX models.

a-c, Percentages of mouse Ter119+CD45- erythroid cells (**a**), mouse CD45+CD11b+Gr-1+ myeloid cells (**b**) and mouse CD41+ MK (**c**) at the treatment endpoint. The percentages of mouse erythroid cells, myeloid cells and MK in n=34 PDX mice (Untreated n=9, 5-AZA n=9, PXS-5505 n=9; P+A n=7) established from n=6 patients were normalized to the percentage of mouse BM fraction (mouse BM fraction [%] = 100% - humanCD45+% - humanCD235a+CD45-% - humanCD41+CD45-%); The data are median±IQR. Kruskal-Wallis test with Dunn's multiple comparisons was used for statistical analysis. Each box represents the IQR and median of the mouse bone marrow fractions in each group. Whiskers indicate Min and Max.

d, e, Changes of mouse hemoglobin (Hb, **d**) level and platelet count (PLT, **e**) during the treatment. The data are presented as mean±SD. Kruskal-Wallis test with Dunn's multiple comparisons was used for comparing Hb or PLT between four arms in each time point.

f, Changes of mouse body weight during the treatment. The data are presented as mean±SD.

P values < 0.05 indicated statistical significance. Source data are provided as a Source Data file.

Supplementary Figure 15

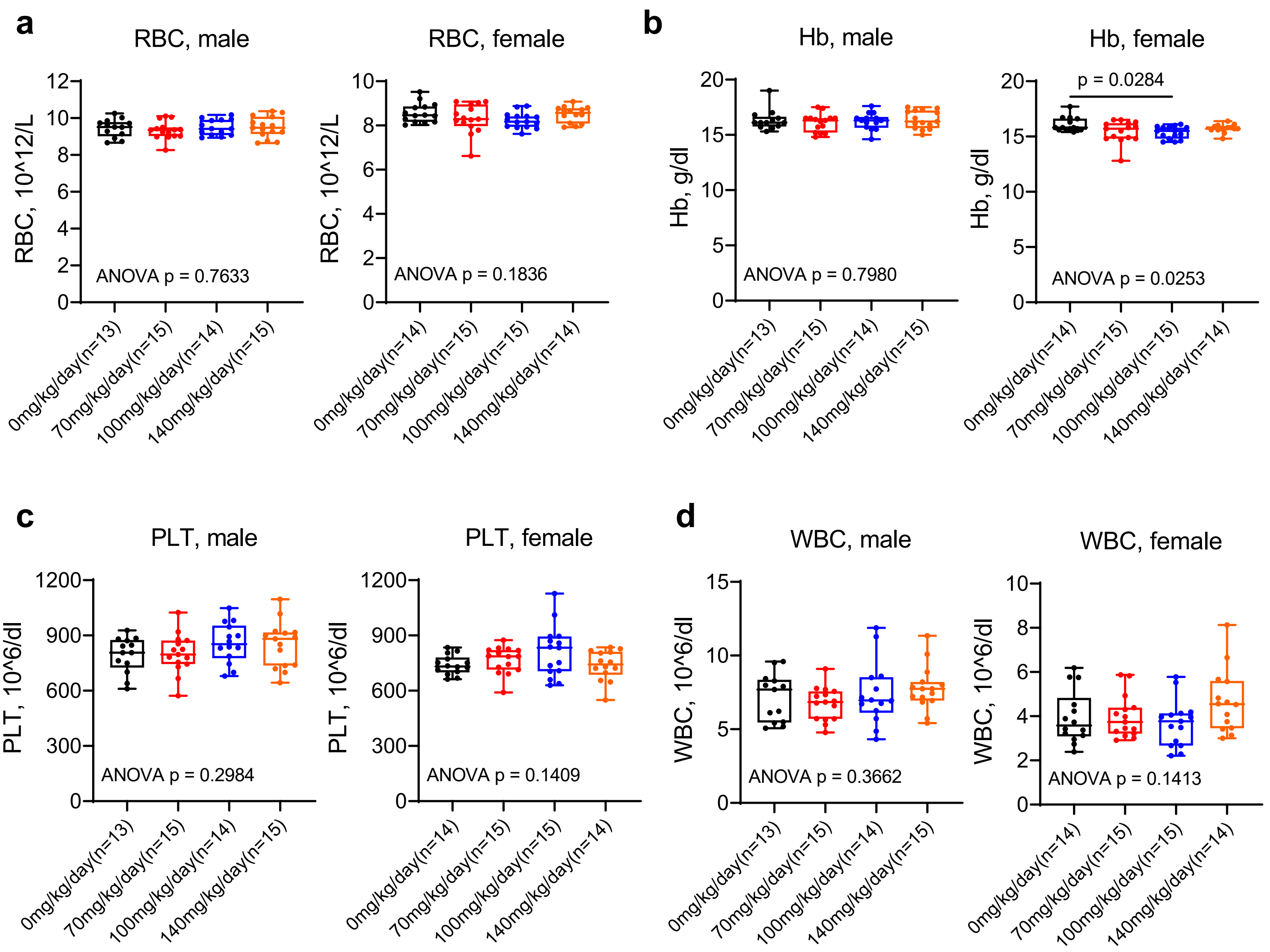

Supplementary Figure 15. The assessment of blood parameters in Wistar Han rats after different doses of oral PXS-5505 treatment.

The data are provided for red blood cell count (RBC, **a**), hemoglobin (Hb, **b**), platelets (PLT, **c**) and white blood cell count (WBC, **d**). The data are median±IQR. Statistical significance was assessed using ordinary one-way ANOVA with Tukey's multiple comparisons. Each box represents the IQR and median of the blood parameters in each group. Whiskers indicate Min and Max. P values < 0.05 indicated statistical significance. Source data are provided as a Source Data file.

Supplementary Figure 16

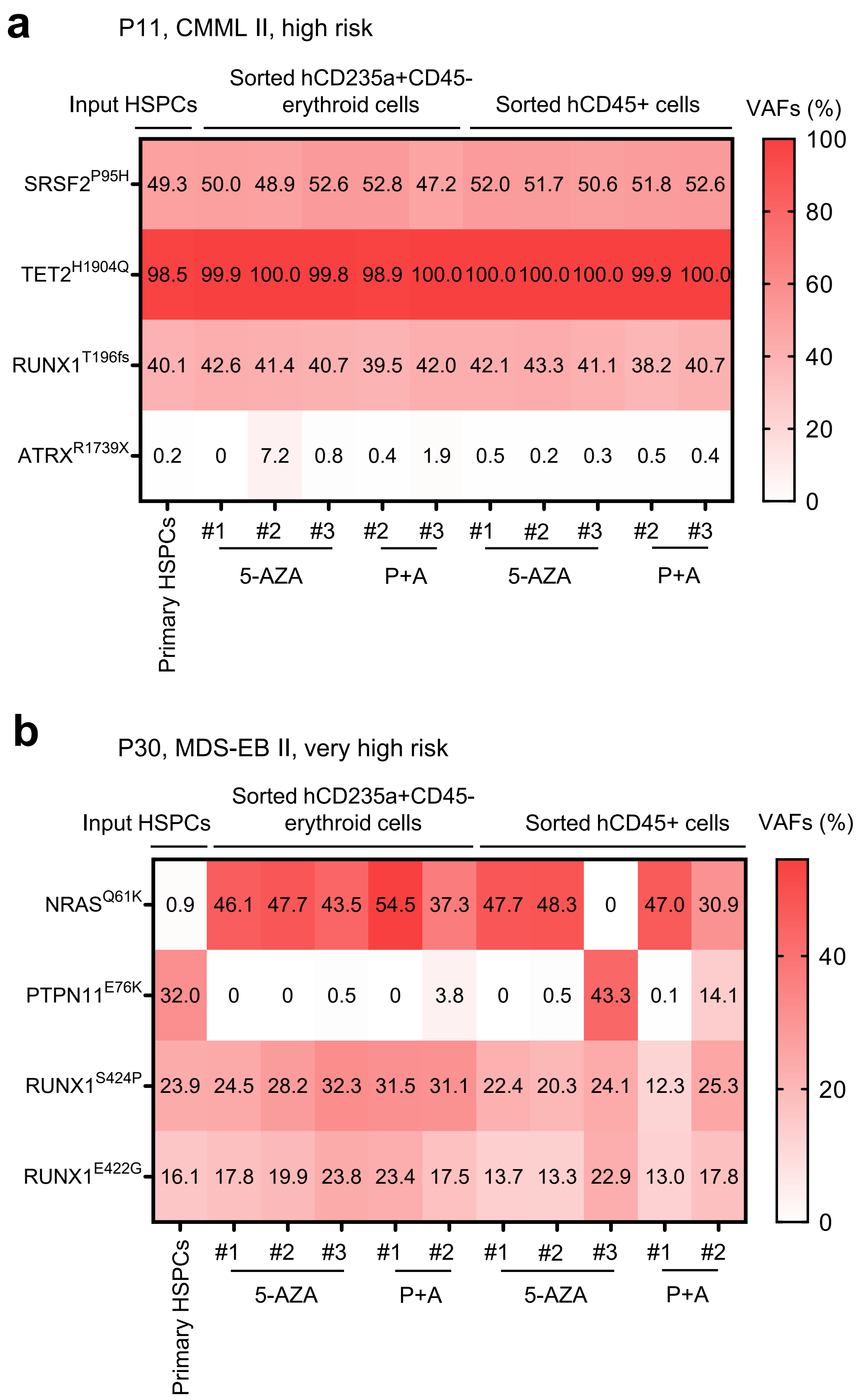

Supplementary Figure 16. Mutational status of human erythroid progenitors sorted from murine PDX model. Mutational status of human erythroid progenitors sorted from murine PDX model of P11 (**a**) and P30 (**b**). Human CD45+ cells and CD235a+CD45- erythroid progenitors were FACS-sorted from PDX models at the treatment endpoint (week 24) and subjected to the panel deep sequencing together with primary MN CD34+ HSPCs. Heatmaps indicate VAFs (%). Source data are provided as a Source Data file.

Supplementary Figure 17

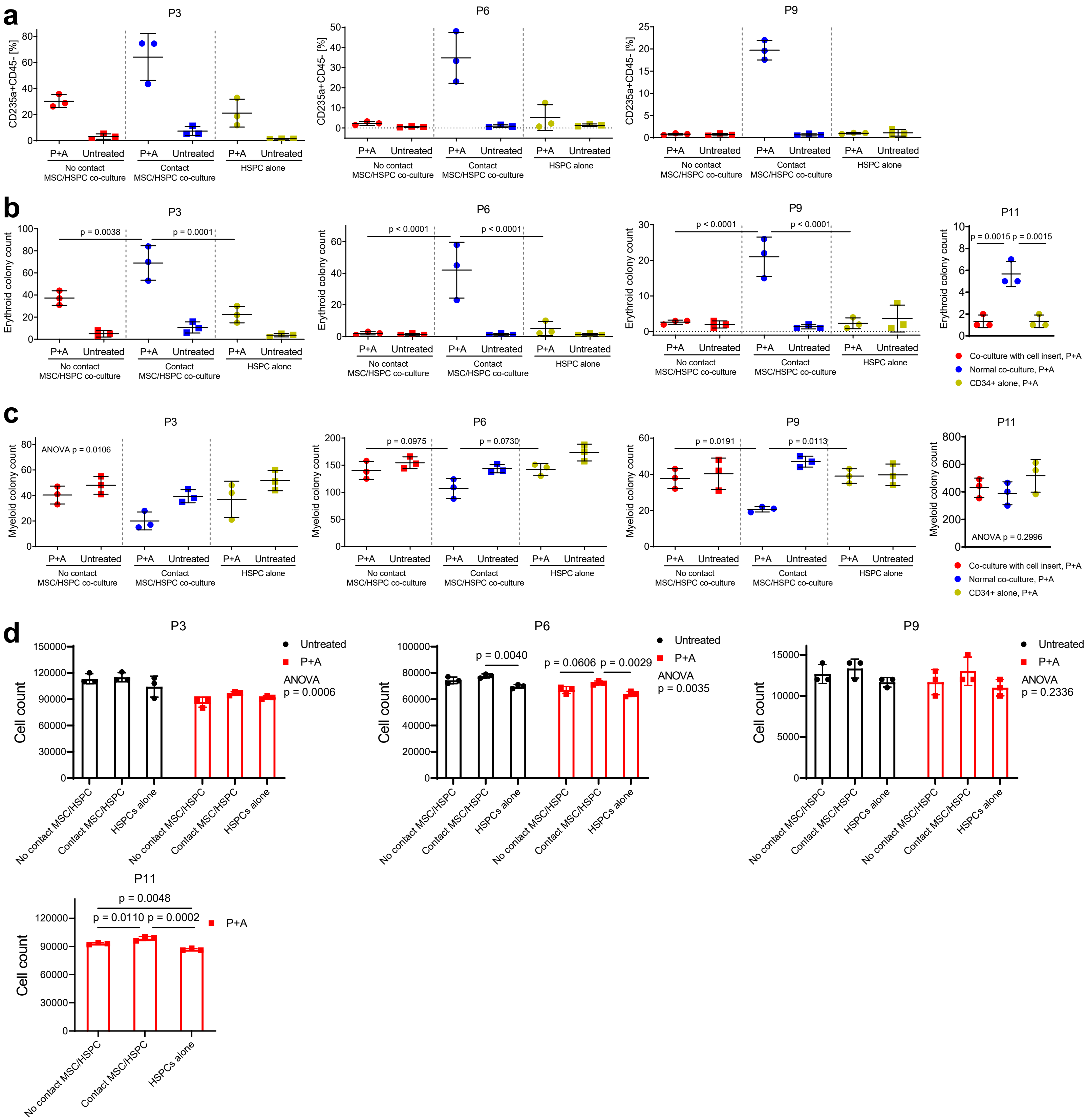

Supplementary Figure 17. MSC/HSPC transwell co-culture. HSPCs of n=3 P+A erythroid responders (P3, P6 and P9) were cultured alone (without MSCs) or co-cultured with autologous MSCs in transwells (non-contact co-culture) or cell-cell contact conditions without treatment or in the presence of P+A.

a-c, Erythroid differentiation (CD235a+CD45-) of HSPCs (**a**), erythroid colony count (**b**) and myeloid colony count (**c**) were assessed after CFU assay. The data are mean±SD of independent co-culture triplicates.

d, HSPC count (HSPC recovery) was assessed after 4 days of MSC/HSPC co-culture. The data are mean±SD of independent co-culture triplicates.

For **b**, **c** and **d**, statistical significance was assessed using ordinary one-way ANOVA with Tukey's multiple comparisons. P values < 0.05 indicated statistical significance. Source data are provided as a Source Data file.

Supplementary Figure 18

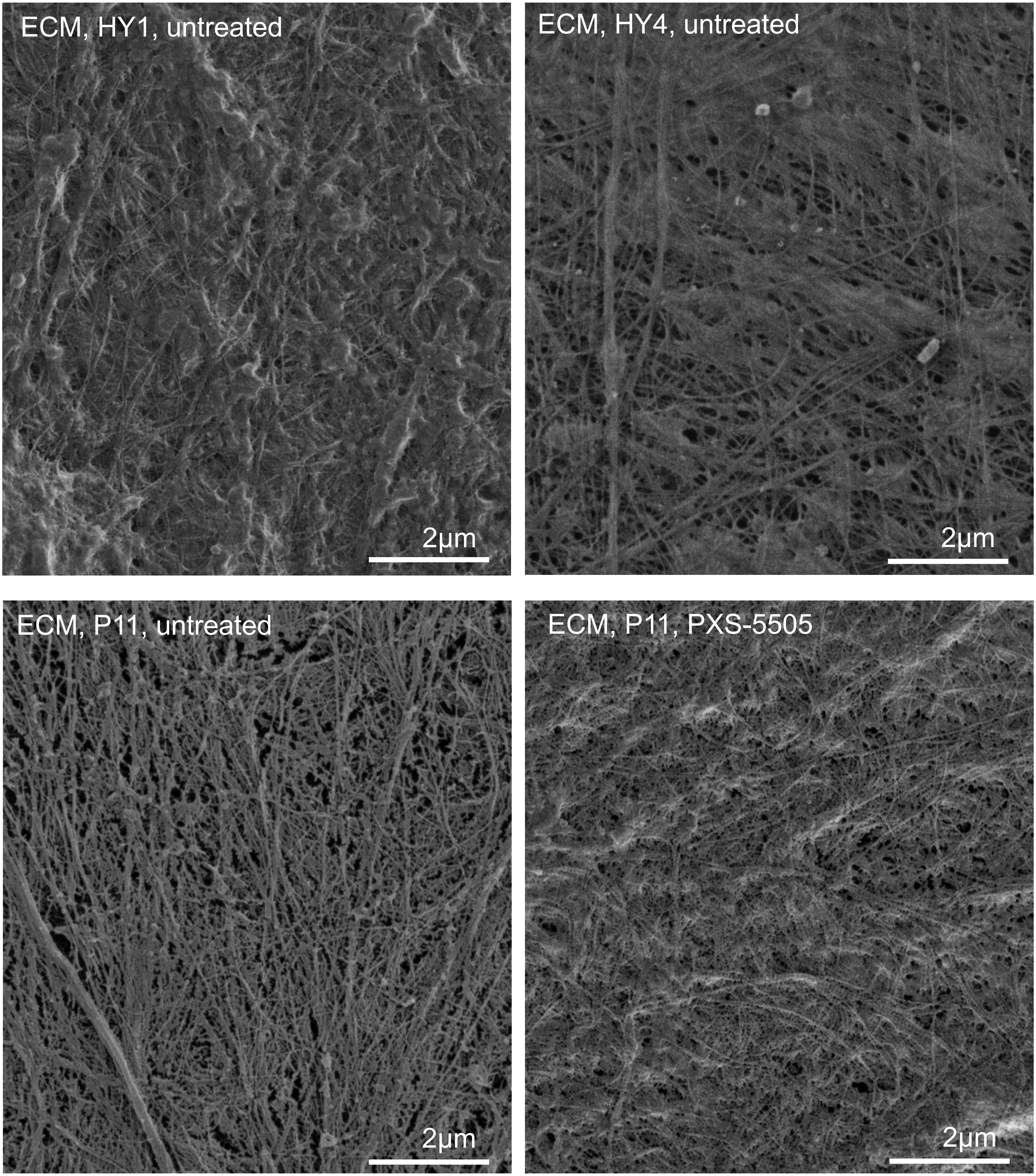

Supplementary Figure 18. Electronic scanning microscopy images of ECM deposited by healthy (HY1 and HY4) and MN MSCs (P11). The experiment was repeated twice with similar results.

Supplementary Figure 19

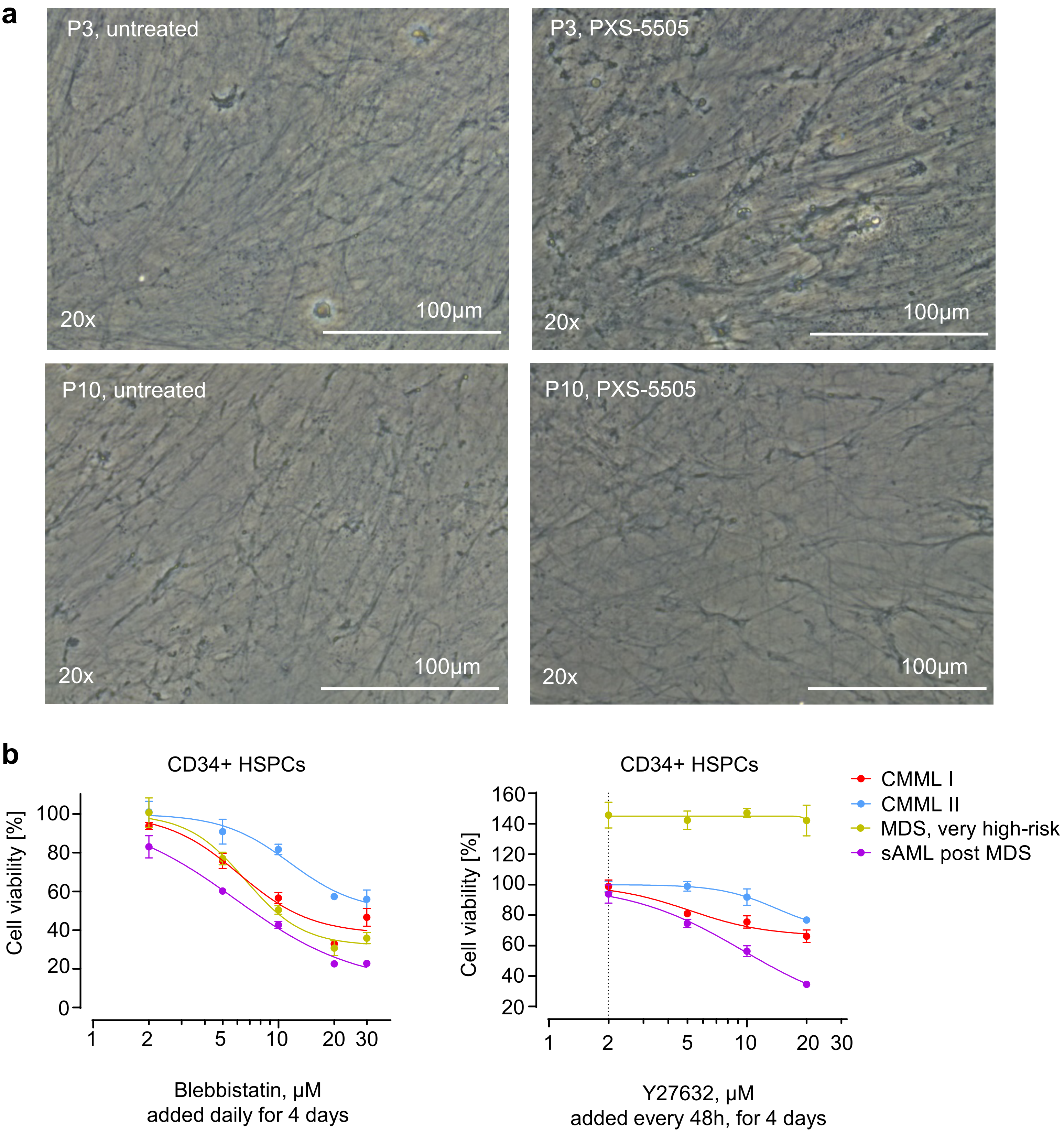

Supplementary Figure 19. P+A induced erythroid differentiation depends on direct contact of CD34+ HSPCs with MSCs-derived ECM.

a, Phase contrast microscopy images of untreated and PXS-5505-treated MSCs derived ECM of n=2 P+A erythroid responders (P3 [top] and P10 [bottom]). The experiment was repeated twice with similar results.

b, Optimization of Blebbistatin and Y27632 concentrations for ECM contact inhibition assay. CD34+ HSPCs of n=4 MN patients were treated with increasing concentrations of Blebbistatin (left) and Y27632 (right) followed by the assessment of cell viability using CellTiter-Glo assay. The data are mean±SD of 6 replicates. Source data are provided as a Source Data file.

Supplementary Figure 20

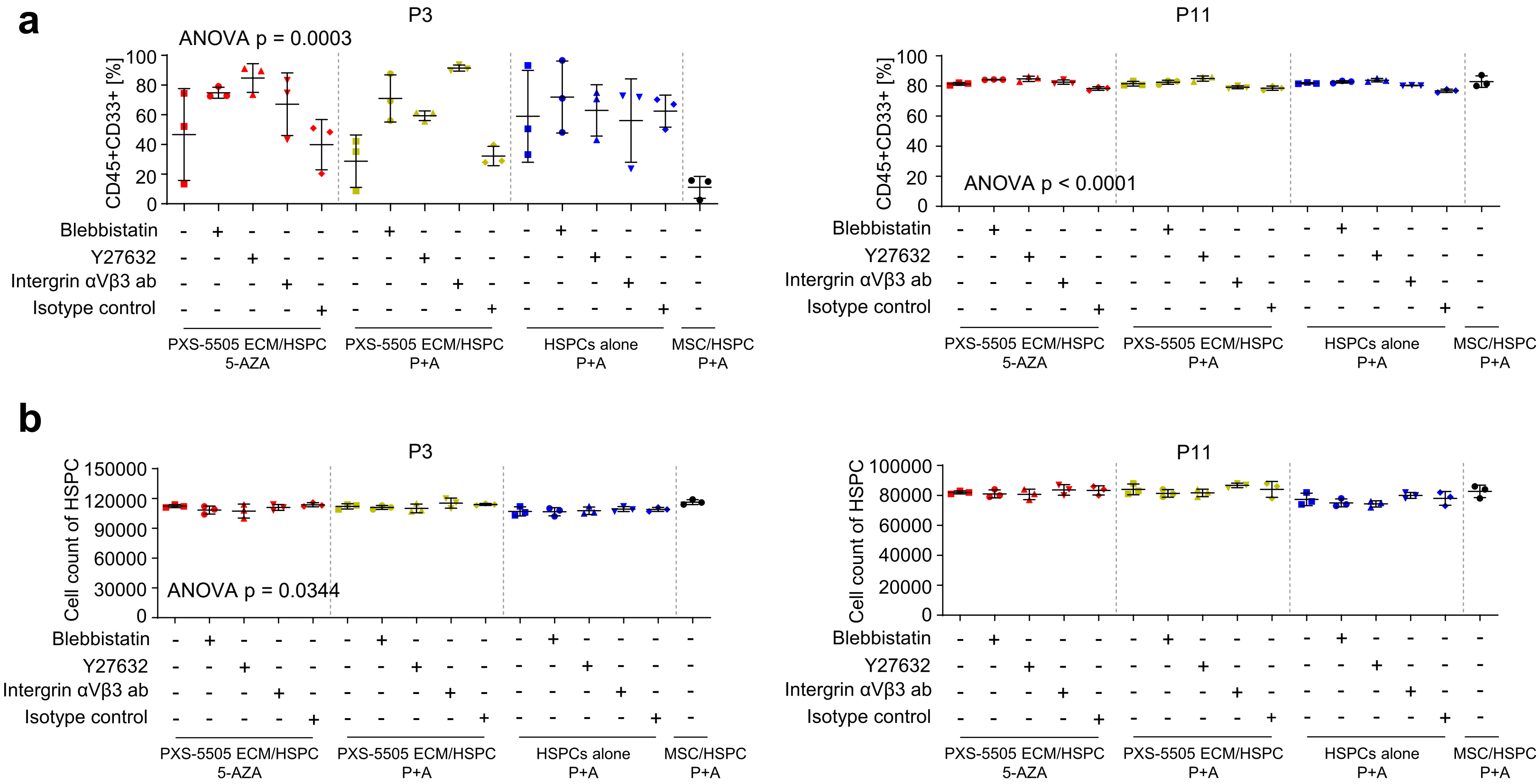

Supplementary Figure 20. The effects of integrin inhibition on the HSPCs differentiation and cell recovery.

CD34+ HSPCs of n=2 MN patients (P3 and P11) were cultured on the PXS-5505 ECM in the presence of 5-AZA, P+A and inhibitors. As a control, HSPCs were cultured without ECM in the presence of inhibitors.

a, Myeloid differentiation (CD45+CD33+ cells) was assessed by flow cytometry after CFU assay.

b, HSPC count (HPSC recovery) was assessed after 4 days of treatment.

For **a** and **b**, the data are mean±SD of independent HSPC/ECM culture triplicates. Statistical significance was assessed using ordinary one-way ANOVA with Tukey's multiple comparisons. P values < 0.05 indicated statistical significance. Source data are provided as a Source Data file.
