# Supplementary material for: Inhibition of lysyl oxidases synergizes with 5-azacytidine to restore erythropoiesis in myelodysplastic and myeloid malignancies
Source: Nat Commun. 2023 Mar 17;14:1497. doi: 10.1038/s41467-023-37175-8 (PMC10023686; doi:10.1038/s41467-023-37175-8)
Supplement: Supplementary file 2 — Reporting Summary [file 41467_2023_37175_MOESM2_ESM.pdf]

nature portfolio

Corresponding author(s): Vladimir Riabov and Daniel Nowak

Last updated by author(s): Feb 15, 2023

Reporting Summary

Nature Portfolio wishes to improve the reproducibility of the work that we publish. This form provides structure for consistency and transparency in reporting. For further information on Nature Portfolio policies, see our Editorial Policies and the Editorial Policy Checklist.

Statistics

For all statistical analyses, confirm that the following items are present in the figure legend, table legend, main text, or Methods section.

n/a	Confirmed	
☐	☒	The exact sample size (*n*) for each experimental group/condition, given as a discrete number and unit of measurement
☐	☒	A statement on whether measurements were taken from distinct samples or whether the same sample was measured repeatedly
☐	☒	The statistical test(s) used AND whether they are one- or two-sided<br>*Only common tests should be described solely by name; describe more complex techniques in the Methods section.*
☒	☐	A description of all covariates tested
☐	☒	A description of any assumptions or corrections, such as tests of normality and adjustment for multiple comparisons
☐	☒	A full description of the statistical parameters including central tendency (e.g. means) or other basic estimates (e.g. regression coefficient) AND variation (e.g. standard deviation) or associated estimates of uncertainty (e.g. confidence intervals)
☐	☒	For null hypothesis testing, the test statistic (e.g. *F*, *t*, *r*) with confidence intervals, effect sizes, degrees of freedom and *P* value noted<br>*Give P values as exact values whenever suitable.*
☒	☐	For Bayesian analysis, information on the choice of priors and Markov chain Monte Carlo settings
☒	☐	For hierarchical and complex designs, identification of the appropriate level for tests and full reporting of outcomes
☐	☒	Estimates of effect sizes (e.g. Cohen's *d*, Pearson's *r*), indicating how they were calculated

Our web collection on statistics for biologists contains articles on many of the points above.

Software and code

Policy information about availability of computer code

Data collection: Fluorescent images were acquired using Leica LAS X 3.4.1.17822 software.

Data analysis: Flowcytometry data was analyzed with the FlowJo software (version 10.5.3)
The images of Gomori silver impregnation staining were quantitatively evaluated by QuPath software (version 0.3.0, https://qupath.github.io/).
The intensity of human mitochondria staining was analyzed using Aperio ImageScope software (Leica Biosystems, version 12.4.3.5008).
For analyzing mass spectrometry (MS) and to generate the ion library, extracted MS/MS spectra were firstly screened against the reviewed Uniprot Human database using the ProteinPilot search engine (AB Sciex, version 5.0.2) accepting cysteine alkylation and common biological modifications. The SWATH acquisition was then performed using looped isolation windows of 20Da. The acquired data were processed with the SWATH Acquisition MicroApp 2.0 in PeakView Software (AB Sciex, version 2.2.0). Protein ion intensity data were imported into MarkerView (AB Sciex, version 1.3.1) to perform most likely ratio (MLR) normalization.
For analyzing next generation sequencing (NSG) data, we used the following publicly available software modules as outlined in the detail in the supplementary method sections:
Trimmomatic (version 0.39);
Seqtk (version 1.2);
FastQC package (version 0.11.5);
Alignment bwa (version 0.7.9);
MarkDuplicates (Picard) (version 2.20.5);
gatk bundle (version 3.8);
bamUtil clipOverlap (version 1.0.14);

Mutect 2 (gatk version 4.1.3.0);
VarScan (version 2.4.4 mpileup 2 snp and mpileup 2 indel);
Sequenza version 4;
SciClone package (version 1.1);
R (version 3.6.3).
Annotated variants obtained in NGS analysis were verified by manual visualization in IG Viewer (version 2.3.98)
The standard statistical analyses were performed using GraphPad Prism 8.4.3 software (San Diego, CA, USA).

For manuscripts utilizing custom algorithms or software that are central to the research but not yet described in published literature, software must be made available to editors and reviewers. We strongly encourage code deposition in a community repository (e.g. GitHub). See the Nature Portfolio guidelines for submitting code & software for further information.

Data

Policy information about availability of data

All manuscripts must include a data availability statement. This statement should provide the following information, where applicable:

- Accession codes, unique identifiers, or web links for publicly available datasets
- A description of any restrictions on data availability
- For clinical datasets or third party data, please ensure that the statement adheres to our policy

The whole exome-sequencing and myeloid panel sequencing data have been deposited in the EGA archive (Accession code: EGAS00001006174). These data are publicly available according to the policy of the EGA platform. Access can be requested via EGA, but also by direct personal communication with the corresponding authors of the manuscript. Responses can be expected within 72 h. Mass spectrometry proteomics data have been deposited to the ProteomeXchange Consortium via the PRIDE partner repository (Accession code: PXD031217). Complete digital scans of mouse femurs as a source data for Figure 4g are available at BioStudies EMBL-EBI portal (Accession code: S-BSST1021). The remaining data are available within the Article, Supplementary Information or Source Data file. Source data are provided with this paper.

Human research participants

Policy information about studies involving human research participants and Sex and Gender in Research.

Reporting on sex and gender	Information on the sex of participants was shown in the text of manuscript and Supplementary Table 2 and 3. Sex based analysis was no performed for this study.
Population characteristics	Characteristics of patients and healthy donors are detailed in Supplementary Tables 1-6 of the manuscript.
Recruitment	The main recruitment criterium was a confirmed diagnosis of myelodysplastic syndrome (MDS), myeloproliferative neoplasms (MPN), MDS/MPN overlap syndrome or secondary acute myeloid leukemia (sAML). As specified in the methods section, biosamples of MN patients were obtained from residual material from diagnostic BM aspirations.<br>The study consisted of n=56 patients with myeloid neoplasms (MN), who were treated at the Department of Hematology and Oncology of the Medical Faculty Mannheim, Heidelberg University, Germany (median age 72.5 years old, range 44-88). As for healthy controls (n=16), hematopoietic cells were obtained from bone specimen from femur endoprosthesis surgery (median age 67 years old, range 51-92). Bone marrow (BM) samples from these patients or healthy controls were used for RT-qPCR, CellTiter-Glo cell viability assay, LOX/LOXL activity inhibition assay, collagen production assessment, co-cultures of mesenchymal stem cell (MSC)/extracellular matrix (ECM) and hematopoietic stem and progenitor cells (HSPC) as well as patient-derived xenograft (PDX) models.<br>In the analysis of the concentration and enzymetic activity of LOX and LOXL2 in BM plasma, n=94 MN patients (median age 73 years old, range 43-88) and n=15 healthy donors (median age 25 years old, range 21-79) were included. These MN patients were also treated at the Department of Hematology and Oncology of the Medical Faculty Mannheim, Heidelberg University, Germany. The BM of young healthy donors (<50 years old) was collected by voluntary iliac crest puncture. The BM from old healthy donors (>50 years old) were obtained as described above.<br>All patients and healthy donors provided written informed consent and all interventions were performed in accordance with the Declaration of Helsinki. There was no bias in selection of patient samples.
Ethics oversight	The use of primary human materials for research purposes was approved by the Medical Ethics Committee II of the Medical Faculty Mannheim of the Heidelberg University.

Note that full information on the approval of the study protocol must also be provided in the manuscript.

Field-specific reporting

Please select the one below that is the best fit for your research. If you are not sure, read the appropriate sections before making your selection.

☒ Life sciences ☐ Behavioural & social sciences ☐ Ecological, evolutionary & environmental sciences

For a reference copy of the document with all sections, see nature.com/documents/nr-reporting-summary-flat.pdf

Life sciences study design

All studies must disclose on these points even when the disclosure is negative.

Sample size	The sample size of involved human biosamples was not pre-determined. Sample size was based on the availability of patient cryopreserved samples stored in the Department of Hematology and Oncology of the Medical Faculty Mannheim, Heidelberg University, Germany. More detailed information of sample size is displayed in supplementary tables 1-6 of the manuscript.<br>In RT-qPCR, we included n=20 MN patients and n=9 healthy donors.<br>In the analysis of the concentration and enzymetic activity of LOX and LOXL2 in BM plasma, n=94 MN patients and n=15 healthy donors were included.<br>In the CellTiter-Glo cell viability assay, samples from n=11 MN patients and n=3 healthy donors were included.<br>In the LOX/LOXL activity inhibiting assay, MSCs from n=5 MN patients were included.<br>In the co-cultures of MSC and HSPC and all colony-forming unit assays, we included a total of n=31 MN patients and n=7 healthy donors.<br>In the PDX models, MSCs and HPSCs from n=6 MN patients were used. A total of n=63 NSG mice were used for PDX model.<br><br>The sample size of n=15 animals per treatment group for the experiment with Wistar Han rats was required to reject the null hypothesis in ANOVA analysis of 4 groups with a power of 0.80 (type II error β=0.2) and type I error α=0.05.
Data exclusions	There was no data exclusion.
Replication	For the experiments in this study, all attempts at replication were successful: In-vitro MSC/HPSC co-culture assays included the following readouts: blinded manual assessment of HSPCs colony formation in colony-forming unit (CFU) assays, flow cytometry assessment of erythroid, myeloid and megakaryocytic differentiation as well as assessment of erythroid progenitors clonality using panel sequencing. For some of MSC/HSPC co-culture or MSC/extracellular matrix culture assays, we counted cell number of HSPCs after co-culture and treatment. These readouts were performed using at least n=3 biological replicates.<br>In-vivo PDX mice were grouped into untreated, 5-AZA, PXS-5505 and 5-AZA+PXS-5505 (P+A) arms. Totally, untreated arm included n=14 PDX mice, 5-AZA arm included n=17 mice, PXS-5505 arm included n=17 mice, and P+A arm included n=15 mice.<br>In summary, we analyzed a sufficient number of MN samples and PDX mice to obtain statistical significance and ensure that the results were reproducible.<br>The effects of the PXS-5505 + 5-AZA combination were studied in patients with myelodysplastic and myeloid malignancies. Synergistic effects of the combination treatment on the erythropoiesis were observed in 11/31 = 35% of patients. To replicate the effects of the combination treatment for each responder or non-responder, critical in vitro experiments were performed using at least n=3 biological replicates (Fig. 2b and 2e, Fig. 3, Fig. 6, Fig. 7b and 7f-h, Fig. 8). In addition, the effects of the combination treatment shown in Fig 2e for P3, P6, P7-P11 were successfully reproduced in independent experiments shown in Fig. 6, Fig. 7f-h and Fig. 8. In vivo xenograft experiments were performed using n=3 mice for each treatment arm unless restricted by the availability of the primary patient material and/or occasional animal death during the treatment course. In several cases, sufficient number of replicates (n=3) was not reached due to the limited number of sorted cells for ex vivo assays (Fig. 5). However, the effects of the combination treatment observed in vitro for P3 and P11 (Fig. 2e) were also reproduced in vivo (Fig. 5a, c, d). Besides, elevated LOX/LOXL activity in patients (Fig. 1c) was reproduced in n=2 experiments and validated in Fig. 1e and 1g. Inhibitory effect of the PXS-5505 on the LOX/LOXL activity was observed in all n=5 patient MSCs samples (Fig. 1j) and validated using recombinant LOXL2 and LOXL3 enzymes (Supplementary Fig. 2d). The effects of the combination treatment on the cross-linked collagen production were assessed in n=2 patients with bone marrow fibrosis (Fig. 1k) and reproduced in n=3 co-culture assays (Supplementary Fig. 2f).
Randomization	Wistar Han rats were randomized on a weight stratified basis using Provantis TM 9.3.1, so that comparable distribution of body weights among groups was achieved after randomization (within ±20% of the mean). Patient samples for PDX studies (n=6) were selected based on the availability of the primary material and successful engraftment in NSG mice (>1% engraftment in the bone marrow before treatment start). For the experiments shown in Figures 6-8 only erythroid responders with sufficient amount of a primary material were selected based on the data shown in Fig. 2e.
Blinding	We performed blinded manual assessment of HSPCs colony formation in CFU assays. The percentage of squares (tiles) containing the areas of fibrosis or intra marrow ossifications based on Gomori silver impregnation staining were calculated using QuPath software by the operator blinded to treatment groups.<br>For other experimental assays with objective experimental readout (flow cytometry, LOX/LOXL activity and viability assays) data analysis was performed by an operator blinded to treatment groups. While evaluating the responses of the animals, the scientists were aware of the treatment history. However, due to the technical objectivity of the endpoints to be examined using instumental methods (clonal evolution using NGS sequencing, engraftment rates and erythroid differentiation by flow cytometry, spleen and body weights etc), it was not possible to introduce a bias because instrument setting were kept the same for all samples.

Reporting for specific materials, systems and methods

We require information from authors about some types of materials, experimental systems and methods used in many studies. Here, indicate whether each material, system or method listed is relevant to your study. If you are not sure if a list item applies to your research, read the appropriate section before selecting a response.

Materials & experimental systems

n/a	Involved in the study
☐	☒ Antibodies
☒	☐ Eukaryotic cell lines
☒	☐ Palaeontology and archaeology
☐	☒ Animals and other organisms
☒	☐ Clinical data
☒	☐ Dual use research of concern

Methods

n/a	Involved in the study
☒	☐ ChIP-seq
☐	☒ Flow cytometry
☒	☐ MRI-based neuroimaging

Antibodies

Antibodies used

anti Ter119-APC, Clone TER-119 (eBioscience, Cat No: 17-5921-82) dilution: 1:20, host species: mouse, application: Flow cytometry
anti Gr-1-BV786, Clone RB6-8C5 (BD Biosciences, Cat No: 740850) dilution: 1:20, host species: mouse, application: Flow cytometry
anti CD11b-APC, Clone M1/70 (Biolegend, Cat No: 101212) dilution: 1:100, host species: mouse and human, application: Flow cytometry
anti CD41-APC, Clone MWReg30 (Biolegend, Cat No: 133914) dilution: 1:20, host species: mouse, application: Flow cytometry
anti CD45-APC-Cy7, Clone 30-F11 (Biolegend, Cat No: 103116), dilution: 1:100, host species: mouse, application: Flow cytometry
anti CD45-PE, Clone HI30 (BD Bioscience, Cat No: 555483), dilution: 1:100, host species: human, application: Flow cytometry
anti CD235a-PerCP-Cy5.5, Clone HI264 (Biolegend, Cat No: 349110), dilution: 1:40 for in-vitro assays, dilution: 1:10 for PDX, host species: human, application: Flow cytometry
anti CD71-PE-Cy7, Clone CY1G4 (Biolegend, Cat No: 334112), dilution: 1:5000, host species: human, application: Flow cytometry
anti CD41-PE-Cy7, Clone HIP8 (Biolegend, Cat No: 303718), dilution: 1:5000, host species: human, application: Flow cytometry
anti CD33-APC, Clone WM53 (Biolegend, Cat No: 303408), dilution: 1:100, host species: human, application: Flow cytometry
anti CD34-FITC, Clone 561 (Biolegend, Cat No: 343604), dilution: 1:100, host species: human, application: Flow cytometry
Fc receptors blocking reagent: human (Miltenyi biotec, Cat No: 130-059-901), dilution: 1:10, application: Flow cytometry
Fc receptors blocking reagent: mouse (Miltenyi biotec, Cat No: 130-092-575), dilution: 1:20, application: Flow cytometry
SYTOX™ Blue Dead Cell Stain (Thermofisher Scientific, Cat No: S34857), dilution: 1:2000, application: Flow cytometry
anti fibronectin Alexa Fluor 488 antibody, Clone FN-3 (ThermoFisher Scientific, Cat No: 53-9869-80), dilution: 10µg/ml, host species: human, application: immunofluorescent (IF) staining
anti collagen type I polyclonal antibody (Sigma-Aldrich, Cat No: AB745), dilution: 1:40, host species: human, application: IF staining
secondary goat anti-rabbit Alexa Fluor 488 IgG (H+L) polyclonal antibody (ThermoFisher Scientific, Cat No: A-11034), dilution: 5µg/ml, host species: Goat/IgG, application: IF staining
anti integrin αVβ3 antibody, Clone LM609, azide-free (Sigma-Aldrich, Cat No: MAB1976Z), dilution: 17µg/ml, host species:
canine, rabbit, avian, monkey, pig, human, chicken, bovine, application: as a single treatment for HPSCs cultured with extracellular matrix (ECM)
mouse IgG1-k negative control, Clone MOPC-21, azide free (Sigma-Aldrich, Cat No: MABF1081Z), dilution: 17µg/ml, application: as a single treatment for HPSCs cultured with ECM
anti mitochondria antibody, Clone 113-1 (Sigma-Aldrich, Cat No: MAB1273), dilution: 1:80, host species: human, application: human mitochondria staining

Validation

We have provided a link for the relevant data sheet for each antibody. The data sheet includes the manufacturer's validations statements, quality control procedures and relevant citations:
anti Ter119-APC, Clone TER-119 (eBioscience, Cat No: 17-5921-82), https://www.thermofisher.com/order/genome-database/dataSheetPdf?producttype=antibody&productsubtype=antibody_primary&productId=17-5921-82&version=260
anti Gr-1-BV786, Clone RB6-8C5 (BD Biosciences, Cat No: 740850), https://www.bdbiosciences.com/en-de/products/reagents/flow-cytometry-reagents/research-reagents/single-color-antibodies-ruo/bv786-rat-anti-mouse-ly-6g-and-ly-6c.740850
anti CD11b-APC, Clone M1/70 (Biolegend, Cat No: 101212), https://d1spbj2x7qk4bg.cloudfront.net/en-us/products/apc-anti-mouse-human-cd11b-antibody-345?pdf=true&displayInline=true&leftRightMargin=15&topBottomMargin=15&filename=APC%20anti-mouse/human%20CD11b%20Antibody.pdf&v=20220831123135
anti CD41-APC, Clone MWReg30 (Biolegend, Cat No: 133914), https://d1spbj2x7qk4bg.cloudfront.net/en-us/products/apc-anti-mouse-cd41-antibody-7592?pdf=true&displayInline=true&leftRightMargin=15&topBottomMargin=15&filename=APC%20anti-mouse%20CD41%20Antibody.pdf&v=20221115073101
anti CD45-APC-Cy7, Clone 30-F11 (Biolegend, Cat No: 103116), https://d1spbj2x7qk4bg.cloudfront.net/en-us/products/apc-cyanine7-anti-mouse-cd45-antibody-2530?pdf=true&displayInline=true&leftRightMargin=15&topBottomMargin=15&filename=APC/Cyanine7%20anti-mouse%20CD45%20Antibody.pdf&v=20220831123135
anti CD45-PE, Clone HI30 (BD Bioscience, Cat No: 555483), https://www.bdbiosciences.com/content/bdb/paths/generate-tds-document.de.555483.pdf
anti CD235a-PerCP-Cy5.5, Clone HI264 (Biolegend, Cat No: 349110), https://d1spbj2x7qk4bg.cloudfront.net/en-us/products/percp-cyanine5-5-anti-human-cd235a-glycophorin-a-antibody-9002?pdf=true&displayInline=true&leftRightMargin=15&topBottomMargin=15&filename=PerCP/Cyanine5.5%20anti-human%20CD235a%20(Glycophorin%20A)%20Antibody.pdf&v=20201224043030
anti CD71-PE-Cy7, Clone CY1G4 (Biolegend, Cat No: 334112), https://d1spbj2x7qk4bg.cloudfront.net/en-us/products/pe-cyanine7-anti-human-cd71-antibody-9328?pdf=true&displayInline=true&leftRightMargin=15&topBottomMargin=15&filename=PE/Cyanine7%20anti-human%20CD71%20Antibody.pdf&v=20221115073101
anti CD41-PE-Cy7, Clone HIP8 (Biolegend, Cat No: 303718), https://d1spbj2x7qk4bg.cloudfront.net/en-us/products/pe-cyanine7-anti-human-cd41-antibody-7114?pdf=true&displayInline=true&leftRightMargin=15&topBottomMargin=15&filename=PE/Cyanine7%

20anti-human%20CD41%20Antibody.pdf&v=20220829083035
anti CD33-APC, Clone WM53 (Biolegend, Cat No: 303408), https://d1spbj2x7qk4bg.cloudfront.net/en-us/products/apc-anti-human-cd33-antibody-877?pdf=true&displayInline=true&leftRightMargin=15&topBottomMargin=15&filename=APC%20anti-human%20CD33%20Antibody.pdf&v=20221202014442
anti CD34-FITC, Clone 561 (Biolegend, Cat No: 343604), https://d1spbj2x7qk4bg.cloudfront.net/en-us/products/fitc-anti-human-cd34-antibody-6035?pdf=true&displayInline=true&leftRightMargin=15&topBottomMargin=15&filename=FITC%20anti-human%20CD34%20Antibody.pdf&v=20220831123135
Fc receptors blocking reagent: human (Miltenyi biotec, Cat No: 130-059-901), https://www.miltenyibiotec.com/upload/assets/IM0001232.PDF
Fc receptors blocking reagent: mouse (Miltenyi biotec, Cat No: 130-092-575), https://www.miltenyibiotec.com/upload/assets/IM0001509.PDF
Both of the two Fc receptors blocking reagents have been validated by the published paper: https://pubmed.ncbi.nlm.nih.gov/34172896/.
SYTOX™ Blue Dead Cell Stain (Thermofisher Scientific, Cat No: S34857), https://www.thermofisher.com/order/catalog/product/cn/en/S34857
anti fibronectin Alexa Fluor 488 antibody, Clone FN-3 (ThermoFisher Scientific, Cat No: 53-9869-80), https://www.thermofisher.cn/order/genome-database/dataSheetPdf?producttype=antibody&productsubtype=antibody_primary&productId=53-9869-80&version=243
anti collagen type I polyclonal antibody (Sigma-Aldrich, Cat No: AB745), https://www.sigmaaldrich.com/DE/en/product/mm/ab745?gclid=CjwKCAjwrZOXBhACEiwA0EoRD6WCuL839Zobo0xU5Ea7nqEPnO4Jnre1Hbyb6xg0xAuWJkJu9JRchBoCmmwQAvD_BwE and https://www.karger.com/Article/FullText/499506
secondary goat anti-rabbit Alexa Fluor 488 IgG (H+L) polyclonal antibody (ThermoFisher Scientific, Cat No: A-11034), https://www.thermofisher.cn/antibody/product/A-11034.html?adobe_mc=MCMID%7C76855803250659711343289055655929660161%7CMCAID%3D2FA6739C0515CE2A-4000095A235B6ECD%7CMCORGID%3D5B135A0C5370E6B40A490D44%40AdobeOrg%7CTS=1614293705&ef_id=CjwKCAjwrZOXBhACEiwA0EoRD5s0wcw6pNY9zBy7mZNuNS9Kv3jtTMiodOe0CPOlaqL83O7LZkAWhhoCOqMQAvD_BwE:G:s&s_kwcid=AL!3652!3!516608152221!!!g!!&cid=bid_pca_aus_r01_co_cp1359_pjt0000_bid00000_0se_gaw_dy_pur_con&gclid=CjwKCAjwrZOXBhACEiwA0EoRD5s0wcw6pNY9zBy7mZNuNS9Kv3jtTMiodOe0CPOlaqL83O7LZkAWhhoCOqMQAvD_BwE
anti integrin αVβ3 antibody, Clone LM609, azide-free (Sigma-Aldrich, Cat No: MAB1976Z), https://www.sigmaaldrich.com/DE/en/product/mm/mab1976?gclid=CjwKCAjwrZOXBhACEiwA0EoRD0ypLZ8VOR9R62TbDY5Ocj6wey3FBfLHN6syKO_fbB1z6gYIMEYFshoC9dEQAvD_BwE
mouse IgG1-k negative control, Clone MOPC-21, azide free (Sigma-Aldrich, Cat No: MABF1081Z), https://www.sigmaaldrich.com/DE/en/product/mm/mabf1081z
anti mitochondria antibody, Clone 113-1 (Sigma-Aldrich, Cat No: MAB1273), https://www.sigmaaldrich.com/DE/en/product/mm/mab1273?gclid=CjwKCAjwrZOXBhACEiwA0EoRD-_auZnjm6zeA_hyxkPjOB_G9azRVN56hqV1t6oRUL6wYQi0s1lq_hoCU64QAvD_BwE and https://link.springer.com/article/10.1007/s00534-009-0070-1

Animals and other research organisms

Policy information about studies involving animals; ARRIVE guidelines recommended for reporting animal research, and Sex and Gender in Research

Laboratory animals	Patient-derived xenograft (PDX) models were established in 8-10 week-old NOD.Cg-Prkdcscid Il2rgtm1Wjl/SzJ (NSG) female mice (Jackson Laboratory, 005557). All NSG mice were housed under specific pathogen-free conditions in an animal facility (Medical Faculty Mannheim, Heidelberg University) at a 12h/12h day/night cycle in individually sterilized ventilated plastic cages with adjusted air temperature (21°C) and 50% relative humidity. All NSG mice were fed ad libitum with a sterilized standard redent diet and free access to sterilized water.<br>For the PXS-5505 toxicity studies in Wistar Han rats, both female and male rats were used at the age of 28-32 weeks.<br>Rats were acclimated at Pharmaron animal facility for 3 weeks prior to treatment. Rats were housed in polycarbonate shoebox cages with corn cob bedding and 12-hour light/12-hour dark cycle. The temperature was set to be maintained between 20-26°C. The humidity was maintained between 40-70%. Rats were fed ad libitum with rodent diet provided by Beijing KeAo Xieli Feed Co., Ltd. Water was provided ad libitum via water bottles. The tap water was filtered through four sequential filters (5-10 µm, 1-4.9 µm, 0.2-0.5 µm, and 0.1-0.19 µm), passed through the ultraviolet sterilization system and filled into the water bottle. The bottle including water was autoclaved before given to the animals.
Wild animals	No wild animals were used in this study.
Reporting on sex	PDX models were performed in female NSG mice, as they are known to show higher success rate of obtaining human engraftment compared to males.<br>For the PXS-5505 toxicity studies in Wistar Han rats the toxicity tests were performed on both females and males and toxicity data were reported separately for both sexes.
Field-collected samples	The study did not involve any sample collected from the field.
Ethics oversight	PDX animal experiments were performed in accordance with institutional guidelines and approved by the state authority Karlsruhe, Germany.<br>The PXS-5505 toxicity studies in Wistar Han rats were done in compliance with the Organization of Economic Cooperation and Development (OECD) Principles on Good Laboratory Practice ENV/MC/CHEM (98) 17 (Revised in 1997, Issued January 1998) and the United States Food and Drug Administration (US FDA) Good Laboratory Practice (GLP) Regulations for Nonclinical Laboratory Studies (21 CFR Part 58) guidelines at Pharmaron, China.

Note that full information on the approval of the study protocol must also be provided in the manuscript.

Flow Cytometry

Plots

Confirm that:

☒ The axis labels state the marker and fluorochrome used (e.g. CD4-FITC).

☒ The axis scales are clearly visible. Include numbers along axes only for bottom left plot of group (a 'group' is an analysis of identical markers).

☒ All plots are contour plots with outliers or pseudocolor plots.

☒ A numerical value for number of cells or percentage (with statistics) is provided.

Methodology

Sample preparation	For collecting cells from CFU assay, bulk colonies from each 35mm dish were resuspended in 12ml PBS with 10% FBS as follows: 3ml PBS with 10% FBS was mixed with MethoCult™ mixture and removed into a 15ml centrifuge tube using a transfer pipette. This step was repeated three more times. The pooled bulk colonies were spun down at 2000rpm for 15 min at 4°C.<br>For collecting cells from megakaryocytic (MK) differentiation assay and ex-vivo erythropoietin (EPO)-induced assay, each well of the 24-well plate for cell culture were washed with 500µl PBS with 10% FBS. This step was repeated for three times. All cells from the same well were combined into a Falcon 5 ml round bottom polystyrene test tube. Samples were spun down at 400g for 5min at 4°C.<br>For testing long-term engraftment at 12 weeks post-transplant, we performed bone marrow (BM) puncture based on intrafemoral injection in PDX mice to collect BM samples. These BM samples were mixed with PBS with 10% FBS. At the treatment endpoint of PDX mice, the bones (tibia, femur and ilium) were crushed for BM extraction. In details, bones were homogenized into 3ml sterile PBS with 10% using a mortar. The homogenized bones were filtered into a 50ml sterile centrifuge tube through a strainer (100µm). The homogenization procedure was repeated until complete decoloration of bone fragments. Both of the types of BM samples were centrifuged at 400g for 5min at 4°C.<br>After centrifugation, supernatants were completely discarded and cell pellets (samples from CFU assay, MK differentiation assay and EPO-induced assay as well as BM from PDX mice) were resuspended in FACS buffer (PBS with 0.4% BSA and 0.02% NaN3). All cell suspensions were incubated for 30 min with Fc receptor blocking reagents and fluorescent dye-labeled antibodies on ice, kept away from light. After incubation, cells were washed once using 1.5ml BD CellWASH with a centrifugation (400g, 5min, 4°C) and resuspended in FACS buffer or sorting buffer (HBSS with 2mM EDTA, 10mM HEPES and 1% FBS) containing SYTOX blue for further test.
Instrument	BD FACSMelody Cell Sorter (FACS), BD FACSAria IIu and FACSAria™ Fusion" Cell Sorters
Software	FlowJo software (version 10.5.3)
Cell population abundance	At least 10000 events were acquired for cells in each assay. For FACS sorting, the sorted cells were reanalyzed to assess purity. More than 75% purity was achieved.
Gating strategy	Cells were initially gated over the 2D density of events on forward and side scatter (FSC-A/SSC-A) to exclude cell debris. Subsequently, live cells were gated on absence of sytox blue staining. Downstream gating is described for each experiment (Supplementary Figures 3, 7, 9, 11, 12 and 13).

☒ Tick this box to confirm that a figure exemplifying the gating strategy is provided in the Supplementary Information.
